# Gamma‐Glutamyl Cysteine Ligase Activity as a Proxy for Human T Cell Function and Drug‐Induced Immunosuppression

**DOI:** 10.1002/advs.202501179

**Published:** 2025-06-30

**Authors:** Francisco Fueyo‐González, Carmen Salto‐Giron, Mehek Ningoo, Laura Espinar‐Barranco, Rafael Salto, Jose Manuel Paredes, Rosario Herranz, Angel Orte, Miguel Fribourg, Juan A. González‐Vera

**Affiliations:** ^1^ Instituto de Química Médica (IQM‐CSIC) Juan de la Cierva 3 Madrid 28006 Spain; ^2^ Department of Pharmacological Sciences Center of Translational Medicine and Pharmacology Icahn School of Medicine at Mount Sinai New York NY 10029 USA; ^3^ Department of Medicine Translational Transplant Research Center Immunology Institute Icahn School of Medicine at Mount Sinai New York NY 10029 USA; ^4^ Nanoscopy‐UGR Laboratory. Departamento de Fisicoquímica Unidad de Excelencia de Química Aplicada a Biomedicina y Medioambiente Facultad de Farmacia Universidad de Granada Campus Cartuja Granada 18071 Spain; ^5^ Departamento de Bioquímica y Biología Molecular II Unidad de Excelencia de Química Aplicada a Biomedicina y Medioambiente Facultad de Farmacia Universidad de Granada Campus Cartuja Granada 18071 Spain

**Keywords:** glutamate‐cysteine ligase, glutathione, immunosuppression, lanthanide sensor, T cells

## Abstract

T cell effector functions are critical for immune defense, but their dysregulation can cause diseases like immune exhaustion in cancer and loss of tolerance in autoimmunity. Curtailing these functions is essential in therapies such as chimeric antigen receptor T‐cell (CAR‐T) therapies or organ transplantation to avoid hyperactivation and rejection. A major challenge in the field is the precise, live measurement of T cell function at the single‐cell level, limiting the prediction of immune responses, the development of effective immunotherapies, and optimization of immunosuppressive regimens. Gamma‐Glutamyl Cysteine Ligase (GCL), the rate‐limiting enzyme in glutathione (GSH) synthesis, is essential for T cell function in mice, but its role in human T cells is underexplored. **GLed**, a novel reversible lanthanide‐based GSH sensor is introduced that enables real‐time, quantitative measurements of GCL activity at single‐cell resolution. The **GLed** approach distinguishes GSH contributions from GCL and GSR, linking GCL activity directly to human T cell effector functions. Additionally, this reveals previously unknown modulation of GCL activity by immunosuppressive drugs, underscoring GCL as a critical player in T cell function and a potential therapeutic target in immune‐related diseases.

## Graphical abstract

**GLed**, a reversible lanthanide‐based GSH sensor that enables real‐time, single‐cell measurement of Gamma–Glutamyl Cysteine Ligase (GCL) activity ‐whose role in human T cells remains unclear‐ is developed. **GLed** directly links GCL activity to T cell effector functions and reveals its modulation by immunosuppressive drugs, identifying GCL as a critical regulator and potential therapeutic target in immune‐related diseases.

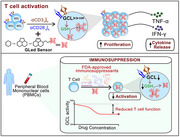

## 1 Introduction

T cell effector functions are essential for immune defense and homeostasis,^[^ ^1^ ^]^ but their dysregulation is linked to various immune‐related diseases. In cancer, T cells can become exhausted,^[^ ^2^, ^3^ ^]^ while in CAR‐T cell therapies, hyperactivation can cause severe toxicities including cytokine release syndrome and immune effector cell‐associated neurotoxicity syndrome.^[^ ^3^, ^4^ ^]^ Autoimmune diseases arise from the loss of tolerance,^[^ ^5^ ^]^ leading to chronic inflammation, while in transplantation, T cells drive graft rejection if immunosuppression is insufficient,^[^ ^6^, ^7^ ^]^ yet excessive suppression increases infection and malignancy risks.^[^ ^8^, ^9^ ^]^ Achieving the right balance of immunosuppression is critical for graft survival and immune stability.

A major challenge in improving outcomes is the lack of real‐time methods to measure T cell functionality and immunosuppression at the single‐cell level. Without precise tools, clinicians rely on indirect markers and generalized dosing, leading to under‐ or over‐suppression. Accurate measurement of T cell states and real‐time tracking of metabolic and enzymatic shifts are crucial for predicting immune responses and developing personalized immunosuppressive therapies.

Gamma–glutamyl cysteine ligase (GCL), the rate‐limiting enzyme in glutathione (GSH) synthesis, plays an essential role in maintaining cellular redox balance and regulating immune cell functions.^[^ ^9^, ^10^, ^11^ ^]^GSH, a key antioxidant thiol, modulates responses to oxidative stress and orchestrates metabolic pathways critical for cellular survival and homeostasis.^[^ ^1^ ^]^ While the role of GCL in T cells has been studied in murine models,^[^ ^12^, ^13^ ^]^ its contribution to human T cell function remains underexplored. Emerging evidence suggests that distinct GSH contributions from GCL and glutathione reductase (GSR) shape immune responses.^[^ ^13^, ^14^, ^15^ ^]^ A significant challenge in studying GCL activity lies in the regulatory complexity introduced by feedback inhibition from GSH, which complicates real‐time monitoring.^[^ ^16^ ^]^ In contrast, GSR activity is more consistently responsive to oxidative stress but also requires sensitive tools for distinction.^[^ ^17^ ^]^ Existing detection methods, predominantly based on fluorescent probes, often lack the sensitivity and reversibility needed to accurately differentiate GSH biosynthesis by GCL from GSR.^[^ ^18^, ^19^, ^20^ ^]^ Moreover, many traditional organic fluorophores suffer from background fluorescence, further limiting their utility in precise and sensitive immunological assays.^[^ ^21^, ^22^ ^]^

Lanthanide‐based luminophores, such as europium (Eu^3^⁺)‐based probes, offer unique advantages, including extended luminescence lifetimes, narrow emission spectra, and resistance to photobleaching.^[^ ^23^, ^24^, ^25^ ^]^ These properties enhance signal‐to‐noise ratios in time‐gated photoluminescence microscopy, addressing the limitations of traditional fluorescent sensors. Despite these advantages, few lanthanide‐based GSH sensors have been developed,^[^ ^26^, ^27^ ^]^ and none have yet demonstrated the ability to differentiate between GCL and GSR contributions in living cells.

Here, we used **GLed**‐based approach, a novel reversible europium‐based luminescent sensor that selectively binds GSH with rapid kinetics and optimal affinity, enabling real‐time, quantitative tracking of GCL activity in live cells. **GLed**’s design, incorporating an europium‐chelating ligand, enhances its stability, cellular uptake, and biocompatibility. Unlike conventional fluorescent probes, it distinguishes GSH synthesized by GCL from that regenerated by GSR, establishing for the first time a direct link between GCL activity and T cell effector functions. Furthermore, our approach revealed a previously unrecognized modulation of GCL activity by immunosuppressive drugs, positioning GCL as both a critical marker of T cell function and a potential therapeutic target in immune‐related diseases. This work provides a versatile approach for dissecting the metabolic and functional dynamics of T cells, with broad implications for autoimmunity, transplantation, and immunotherapy.

## 2 Results and Discussion

### 2.1 Design of Reversible Lanthanide‐Based GSH Sensors

We have recently reported the small molecule 8‐methoxy‐2‐oxo‐1,2,4,5‐tetrahydrocyclopenta[*de*]quinoline‐3‐carboxylic acid that is a reactive non‐fluorescent Michael acceptor, which becomes fluorescent after reaction with thiols.^[^ ^28^ ^]^ In addition, this sensor dynamically self‐assembles with the lanthanide ion Eu^3+^ in water and acts as an efficient Eu^3+^ antenna. Our tetrahydrocyclopenta[*de*]quinoline derivative behaves as a highly selective GSH biosensor, which has demonstrated high potential for studies of metabolism changes in cells of the immune system by using flow cytometry. However, this sensor still presents several limitations for advancing deeper studies in primary cells: i) the lanthanide emission is governed by the dynamic association of free Eu^3+^ ions with the antenna, which can lead to lanthanide release within cells, reducing the efficiency of the sensing, and ii) the response of the sensor is dependent on the capacity of the target cells of uptaking Eu^3+^ cations.

**Figure 1:**
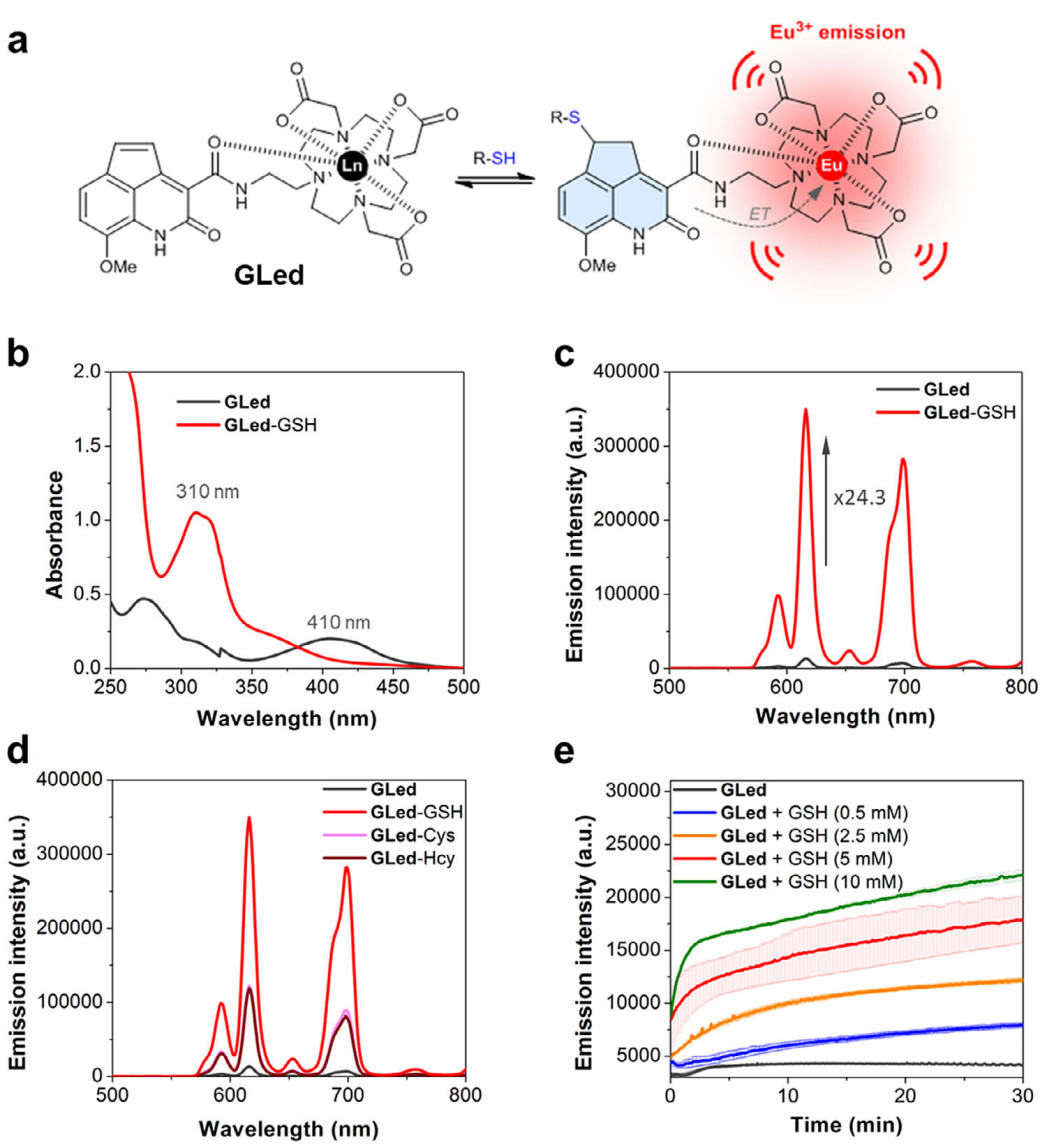
**GLed** behaves as a selective and sensitive GSH sensor. a) Schematic representation of the GSH lanthanide‐based sensor **GLed**. After the addition of GSH to the Michael acceptor (**GLed**), the resulting antenna will become fluorescent and intramolecularly transfer its energy (ET) to the lanthanide ion, leading to a significant increase in its luminescence emission. b) Absorption and c) Representative TG emission spectra (λ_ex_ = 337 nm) of **GLed** (5 µm) before and after reaction with GSH (10 mm) in HEPES buffer at pH 7.4 for 3 h. d) Eu^3+^ luminescence of **GLed** (5 µm, λ_ex_ = 337 nm) upon reaction with GSH, Hcy or Cys (2000 equivalents) in HEPES buffer at pH 7.4 for 3 h. e) Changes in the Eu^3+^ luminescence emission intensity of **GLed** (5 µm) at 616 nm (λ_ex_ = 337 nm) over time, after the addition of GSH (0.5, 2.5, 5, and 10 mm). Shaded areas indicate standard deviation from the average over three repetitions.

To strengthen the binding of the GSH‐sensing moiety to the cation Eu^3+^, thus increasing its stability to avoid the possible cytotoxicity of free Eu^3+^ and facilitating its cell uptake,^[^ ^29^ ^]^ herein we have employed a new molecular design to maximize lanthanide emission in imaging and cytometry for studying GSH dynamic control in T cells. This design involves linking the ligand DO3A (1,4,7,10‐tetraazacyclododecane‐1,4,7‐triacetic acid) to the antenna through an amide bond to obtain the GSH lanthanide‐based sensor (**Figure** **1**a). The use of multidentate ligands offers protection of the metal center from solvent coordination,^[^ ^30^ ^]^ strengthening the antenna/lanthanide binding through high thermodynamic stability,^[^ ^31^, ^32^ ^]^ attenuation of lanthanide‐related cell toxicity and increased cell uptake when used in biological imaging.^[^ ^29^, ^30^, ^35^, ^36^, ^37^, ^38^, ^40^ ^]^ The 2,2′,2′'‐(10‐(2‐(8‐methoxy‐2‐oxo‐1,2‐dihydrocyclopenta[de]quinoline‐3‐carboxamido)ethyl)‐1,4,7,10‐tetraazacyclododecane‐1,4,7‐triyl)triacetic acid^[^ ^28^ ^]^ was obtained by HATU/HOBt/DIPEA mediated coupling of the 8‐methoxy‐2‐oxo‐1,2‐dihydrocyclopenta[de]quinoline‐3‐carboxylic acid with DO3A^t^Bu‐NEtNH_2_ in DMF, followed by hydrolysis of the resulting triester with TFA. Finally, the conjugate was dissolved in HEPES buffer pH 7.4 in the presence of the corresponding lanthanide trichloride salt (TbCl_3_, EuCl_3_, SmCl_3_ or DyCl_3_) to yield the desired DO3A chelates (additional details in Scheme S1, Supporting Information). We tested these lanthanide chelates, including the four lanthanide ions that typically have the most effective emissive transitions (Eu^3+^, Tb^3+^, Sm^3+^ and Dy^3+^), seeking for maximized sensitization of the luminescence of the lanthanide ion response, upon reacting with GSH. Out of the four derivatives, only the one containing Eu^3+^ exhibited a significant luminescence increase (24.3 ± 1.7‐fold, averaged over 4 repetitions) upon reaction with GSH (2000 equivalents) in HEPES buffer at pH 7.4 for 3 h (see Figure S1, Supporting Information for details). These results prompted us to focus on the Eu^3+^‐based GSH detection sensor, which hereafter we call GSH Luminescent europium detection (**GLed**) (Figure 1a).

### 2.2 **GLed** Behaves as a Selective and Sensitive GSH Sensor

The reaction of **GLed** with GSH was evident in the absorption spectrum, which underwent a hypsochromic shift of ≈100 nm, peaking at 310 nm with a broad shoulder up to 400 nm (Figure 1b), due to decreased conjugation on the chromophore moiety. Regarding the emission properties, the reaction with GSH led to sensitization of the luminescence of Eu^3+^ (λ_ex_ = 337 nm) and the appearance of the ^5^D_0_ →^7^F_2_ and ^5^D_0_ →^7^F_4_ bands at 615 and 695 nm, respectively (Figure 1c).

We then tested the capability of **GLed** to discern between GSH and other intracellular thiols such, as cysteine (Cys) and homocysteine (Hcy). For this purpose, we also studied the reactivity of **GLed** (5 µm) toward 2000 equivalents of Cys and Hcy for 3 h in HEPES buffer (50 mm, pH 7.4) and obtained time‐gated (TG) emission spectra and luminescence lifetimes (*τ*) of the Eu^3+^ emissions (see Supporting Information for instrumentation details). In contrast with the high increase in luminescence obtained for GSH (24.3 ± 1.7‐fold, *n* = 4), Cys and Hcy only elicited a 9.8 ± 0.7‐fold (*n* = 4) and 7.2 ± 1.0‐fold (*n* = 4) increase in luminescence, respectively (Figure 1d), thus highlighting the selectivity of our sensor for GSH. Consequently, luminescence lifetimes (*τ*) of the Eu^3+^ emissions of **GLed** in the presence of GSH (679 ± 18 µs, *n* = 6) was higher than the ones in the presence of Hcy (548 ± 9 µs, *n* = 6), Cys (446 ± 18 µs, *n* = 6) and in the absence of biothiols (565 ± 4 µs, *n* = 6). We hypothesize that the selectivity of our probe for GSH is due to a more effective protection against quenching provoked by water molecules compared to the lanthanide complexes of the addition products of Hcy and Cys.^[^ ^40^ ^]^ This enhanced protection of the lanthanide ion in the presence of GSH is likely facilitated by the carboxylate group of the Glu residue present in GSH, which may promote the formation of an extended coordination cage around the ion.^[^ ^40^, ^41^, ^42^ ^]^ Additionally, given the much higher intracellular physiological levels of GSH (1–10 mm) compared to those of Cys or Hcy (30–200 µm),^[^ ^43^, ^44^, ^45^ ^]^ the interfering effects of these thiol‐containing molecules in subsequent cell studies are expected to be negligible. Of note, no significant changes were detected when other potential interfering species (SH_2_, Fe^2+^, Na_2_S_2_O_3_, HClO and NaNO_2_) were studied (Figure S2, Supporting Information).

We next evaluated the kinetics of the response of **GLed** (5 µm) to increasing concentrations of GSH (100, 500, 1000, and 2000 equivalents) over 3 h in HEPES buffer 50 mm at pH 7.4 (Figure 1e). The increase in Eu^3+^ luminescence was dependent on the concentration used. Interestingly, at any studied concentration, inspection of the time course of the luminescence response showed that **GLed** exhibited two different behaviors: an initial fast and immediate reactivity with GSH in the first minute, increasing up to 15‐fold at highest concentration (2000 equivalents) followed by a switch to a slower kinetic phase, which saturated at 1 h (24.3 ± 1.7‐fold increase). These biphasic kinetics indicate that the underlying mechanism of the probe's response is more complicated than the simplified model depicted in Figure 1a. The main reason behind a complex, reversible mechanism may be related to the superposition of additional chemical equilibria in GSH. GSH can exist in reduced and oxidized (disulfide dimer) forms, as well as in five different prototropic forms depending on the pH and coordinating metal cations present in the buffer. Given our objective is designing a reversible probe for *in cellulo* applications, developing a comprehensive kinetic analysis is beyond the scope of this study.

Importantly, the increase in Eu^3+^ luminescence showed a linear response with the GSH concentration in the physiological range of 1–10 mm (Figure S3, Supporting Information). Moreover, **GLed** exhibited an extremely sensitive response to sub‐mm levels of GSH. These results demonstrate the quantitative power of **GLed** for physiologically relevant GSH monitoring. Together with **GLed**’s unique photophysical properties detailed below, it notably exhibited a fast reactivity comparable to the most rapidly responding GSH sensors up to now described in the literature.^[^ ^50^ ^]^

### 2.3 **GLed** Demonstrates Immediate Reversibility, Capturing fast GSH Dynamic Changes in Vitro

**Figure 2:**
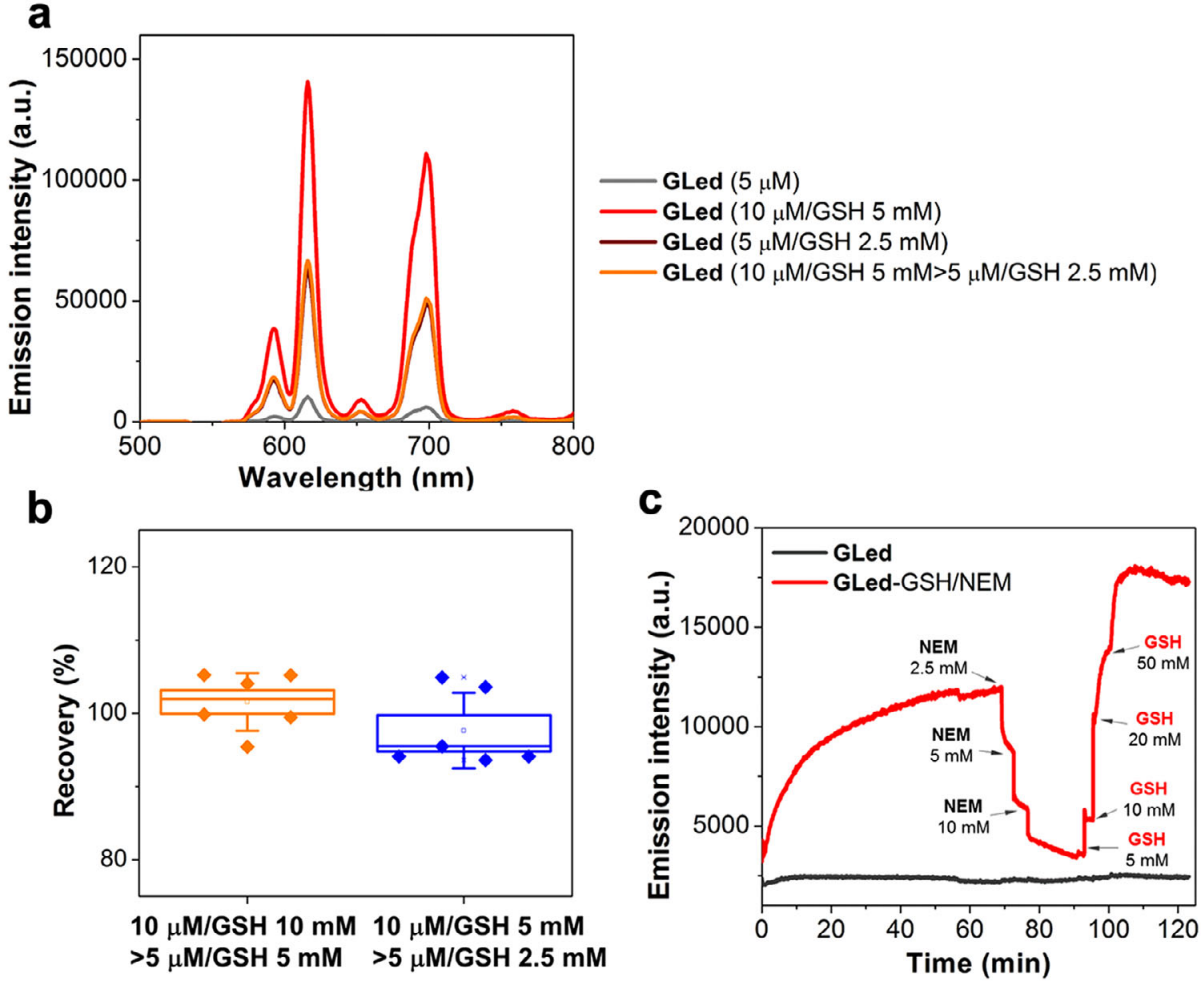
GLed demonstrates immediate reversibility, capturing fast GSH dynamic changes in vitro. a) Representative TG emission spectra of a solution of **GLed** (10 µm) and GSH (5 mm) in HEPES buffer at pH 7.4, diluted two‐fold, resulting in intensity values comparable to those obtained from a **GLed** (5 µm) solution upon the addition of GSH (2.5 mm). b) Recovery of luminescent signal (%) from a two‐fold dilution of a solution containing **GLed** (10 µm) and GSH (5 or 10 mm). c) Demonstration of reaction reversibility between **GLed** and GSH. To a **GLed** (5 µm) solution pre‐incubated with GSH (500 µm) for 1 h, *N*‐ethylmaleimide (NEM, 2.5, 5 and 10 mm) was sequentially added at different times to scavenge all the added GSH, followed by a rapid and complete recovery of the luminescence upon the subsequent addition of increasing concentrations of GSH (5, 10, 20 and 50 mm). The luminescence was collected at 616 nm (λ_ex_ = 337 nm).

To demonstrate the reversibility of the reaction between GSH and **GLed**, we performed dilution experiments combined with “passive” scavenging of GSH with *N*‐ethylmaleimide (NEM), a well‐known irreversible thiol scavenger, as previously established.^[^ ^50^ ^]^ First, a 2‐fold dilution of a solution containing known concentrations of **GLed** (10 µm) and GSH (5 or 10 mm) promptly yielded the same intensity values that those obtained for a solution of **GLed** (5 µm) upon the addition of GSH (2.5 or 5 mm) (**Figure** **2**a; Figure S4, Supporting Information), indicating a recovery efficiency of 101.6 ± 3.9% and 97.6 ± 5.2% for 2.5 and 5 mm GSH, respectively (Figure 2b). This result clearly indicated that the reversible reaction between GSH and **GLed** was extremely rapid and primarily influenced by the concentration of GSH. To further illustrate the reversibility of the reaction, we monitored changes in Eu^3+^ luminescence (λ_ex_ = 337 nm) over time upon the addition of GSH to **GLed**, and then followed by NEM.^[^ ^50^ ^]^ As shown in Figure 2c, the time‐dependent luminescence intensity of a solution of **GLed** (5 µm) pre‐incubated with GSH (500 µm) for 1 h immediately decreased after the addition of increasing concentrations of NEM (2.5, 5 and 10 mm) at different times, followed by a rapid and complete recovery of the luminescence upon the subsequent addition of increasing concentrations of GSH (5, 10, 20 and 50 mm). Overall, these results confirmed the fast reversibility of our sensor, validating it as a sensitive powerful tool to track rapid changes in GSH levels in real‐time in vitro.

### 2.4 **GLed** Monitors GSH Dynamic Changes Mediated by GCL in Cells with High Precision Using Time‐Gated (TG) and Confocal Imaging Luminescence Microscopy

**Figure 3:**
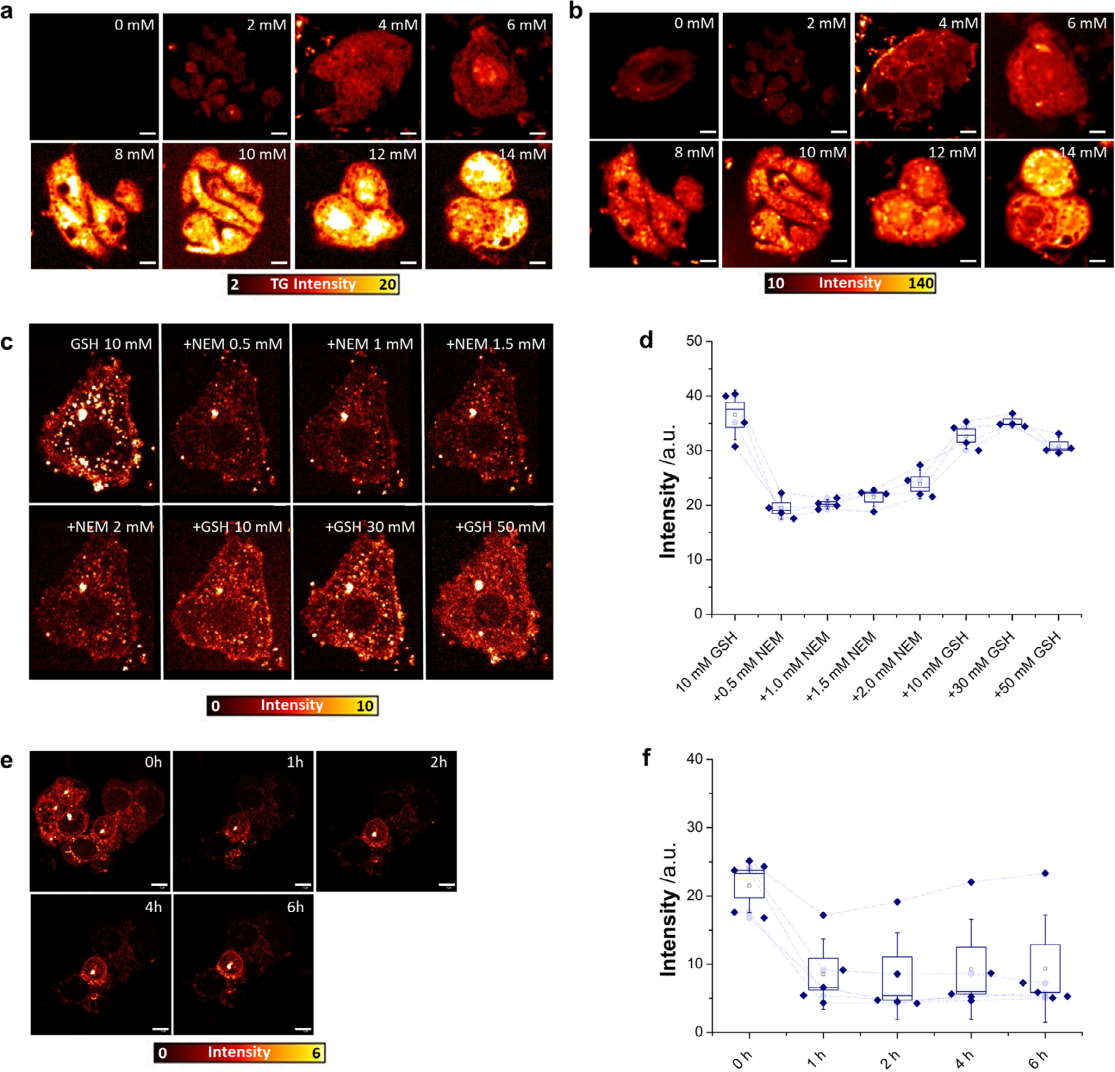
**GLed** monitors GSH dynamic changes mediated by GCL in cells with high precision using TG and confocal imaging microscopy. a) TG and b) confocal PL intensity images of HepG2 cells incubated with **GLed** (5 µm) upon the addition of increasing concentrations (2‐14 mm) of external GSH‐OEt after treatment with NEM. c,d) Real‐time monitoring of GSH level fluctuations in HepG2 cells incubated with **GLed** (10 µm), upon the addition of increasing concentrations of NEM, followed by the addition of increasing concentrations of GSH‐OEt. Boxes in d represent s.d., *n* = 4. The signal variation of individual cells is shown as dashed lines in panel d. e,f) Real‐time monitoring of GSH level fluctuations in HepG2 cells equilibrated with **GLed** (10 µm), upon the addition of the GCL‐selective inhibitor BSO (1 mm). Boxes in f represent s.d., *n* = 5. The signal variation of individual cells is shown as dashed lines in panel f, evidencing that one cell exhibited less response towards BSO and higher GSH levels. In all imaging panels (a,b,c,e), the scale bars represent 5 µm. TG imaging and confocal microscopy were performed with a 375 nm excitation laser using a 580–630 nm bandpass detection filter for Eu^3+^.

The fast reaction kinetics of **GLed** with GSH, in both forward and reverse directions, prompted us to use it for real‐time monitoring of the dynamic changes of GSH in living cells. We used HepG2 cells, a hepatocarcinoma cell line known to generate high levels of GSH (liver cells are strong producers of GSH given the detoxifying role of this organ).^[^ ^50^ ^]^ Initially, the cytotoxicity of **GLed** was evaluated by an MTT assay.^[^ ^50^ ^]^ The results clearly demonstrated that the sensor exhibited extremely low cytotoxicity, making it suitable for long‐term monitoring of GSH dynamic variations. (Figure S5, Supporting Information). To carry out these experiments, the cells were pretreated with NEM (500 µm) to eliminate the endogenous GSH and incubated for 1 h with **GLed** (5 µm) in Hepes (50 mm, pH 7.4). We then added increasing concentrations (2–14 mm) of external glutathione monoethyl ester (GSH‐OEt), a cell‐permeable GSH precursor, to the culture and monitored the Eu^3+^ luminescence emission intensity by TG imaging luminescence microscopy (**Figure** **3**a; Supporting Information for details). This strategy enables enhanced signal‐to‐noise imaging owing to the long luminescence lifetime values of Eu^3+^.^[^ ^51^ ^]^ As shown in Figure 3a the PL intensity of **GLed** significantly increased immediately after the external addition GSH‐OEt to the cells in a concentration‐dependent manner, reaching a maximum value (10‐fold luminescence increase vs the control without GSH‐OEt) at 10 mm of GSH‐OEt. Moreover, despite the advantages of TG microscopy for quantitative biological imaging, the exceptional characteristics of the new sensor enable the rapid measurement of dynamic changes in GSH levels *in cellulo* by using continuous intensity measurements (Figure 3b), which also ensures compatibility with standard flow cytometry experiments.

We next tested whether the fast‐reversible kinetics of **GLed** with GSH observed in our biochemical in vitro assays took place in live cells. To this end, we carried out up‐and‐down kinetic experiments in HepG2 cells incubated with **GLed** (10 µm), monitoring in real‐time the changes in PL intensity upon the addition of increasing concentrations of NEM (0.5, 1, 1.5 and 2 mm), followed by increasing concentrations of GSH‐OEt (10, 30 and 50 mm). As shown in Figure 3c,d, the intensity of the luminescence of **GLed** in HepG2 cells pre‐incubated with GSH‐OEt (10 mm) for 1 h immediately decreased (2.1‐fold) after the addition of increasing concentrations of NEM at different times, followed by a rapid and complete recovery of the luminescence with increasing concentrations of GSH‐OEt (5, 10, 20 and 50 mm). Remarkably, the intensity of the sensor fully recovered the initial values, prior to the addition of NEM. We next asked whether the sensor could detect the impairment of *de novo* GSH synthesis in the cell through inhibition of GCL, which should result in reduced levels of GSH.^[^ ^52^, ^53^, ^54^, ^55^, ^56^, ^57^ ^]^ To test this, we first equilibrated HepG2 cells with **GLed** (10 µm) for 1 h, and then added to the cells L‐Buthionine‐(S,R)‐sulfoximine (BSO, 1 mm), a GCL‐selective inhibitor.^[^ ^58^ ^]^ As predicted, the PL intensity of sensor **GLed** exhibited a clear drop during the first hour and was stable at such low levels for 6 h (Figure 3e,f), supporting the combined use of **GLed** and a BSO to study GCL activity in cells.

### 2.5 **GLed**‐based Approach Tracks GSH Biosynthesis and its Production by GCL in Human T Cells at Single‐Cell Resolution

Our understanding of the role of GSH homeostasis in T cell function is largely based on murine studies, through the ablation of GCL (*de novo* synthesis) and GSR (recycling), the two enzymes responsible for producing GSH.^[^ ^12^, ^13^ ^]^ In mice, GCL, but not GSR, is critical in managing the redox balance in T cells, driving T cell proliferation and effector functions by maintaining GSH biosynthesis.^[^ ^12^, ^13^ ^]^ However, the role of these enzymes in human T cells remains unknown, in part, due to the absence of reversible sensors with appropriate properties to measure GSH dynamic changes at high resolution in living cells. Due to its unique properties, **GLed** is ideally suited to address these challenges and elucidate their contribution to GSH biosynthesis in human T cells.

**Figure 4:**
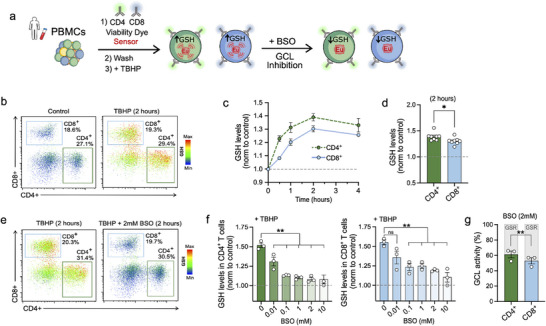
GLed tracks GSH biosynthesis and its production by GCL in human T cells at single‐cell resolution. a) Scheme of the experimental design diagram. b) Representative scatter plots of the GSH levels (overlay) in CD4^+^ and CD8^+^ T cells at single‐cell resolution (CTRL and TBHP). c) Time‐course curves (0‐4 h) of the increase of the GSH levels after adding TBHP followed through the intensity of **GLed** in CD4^+^ (green) and CD8^+^ (blue) T cells (mean ± S.E.M., *n* = 4 per group, three independent experiments). d) Summary bar graphs normalized to control at 2 h (gray line indicates no TBHP control) (mean ± S.E.M., *n* = 8 per group, three independent experiments, *t*‐test compared to control, ^*^ *p*<0.05). e) Comparative graph bars of the GSH levels following 2 h of stimulation with TBHP and subsequent treatment with BSO at increasing concentrations (0–10 mm) (CD4^+^: green and CD8^+^: blue) (mean ± S.E.M., *n* = 3 per group, three independent experiments, ANOVA with post‐hoc Tukey HSD test, ^**^ *p*<0.01). f) Representative scatter plots of the GSH levels (overlay) in CD4^+^ and CD8^+^ T cells at single‐cell resolution (TBHP and TBHP + BSO (2 mm) at 2 h, and g) bar summary of average GCL activity (%) (mean ± S.E.M., *n* = 3 per group, two independent experiments, *t*‐test, ^**^ *p*<0.01).

We first ruled out any deleterious effects of **GLed** on T cell viability; **GLed** exhibited no toxicity at a wide range of concentrations (0–50 µm) after 24 h incubation (Figure S6a, Supporting Information). To demonstrate the sensor's ability to capture dynamic changes in GSH production in human T cells over time, we stimulated peripheral blood mononuclear (PBMCs, which include lymphocytes, such as T cells, B cells, and NK cells, as well as monocytes and dendritic cells) with a low dose of tert‐butyl hydroperoxide (3.5 µm, TBHP) (**Figure** **4**a), an inducer of oxidative processes in cells and tissues commonly used to investigate cellular responses to oxidative stress. After staining for CD4^+^ and CD8^+^ T cell markers, we measured the overall GSH production in these two subpopulations over a period of 0–4 h with TBHP using flow cytometry (Figure 4b). GSH levels increased in both CD4^+^ and CD8^+^ T cells over untreated controls as early as 30 min post‐stimulation, reaching peak levels at 2 h (40% increase for CD4^+^, 30% increase for CD8^+^) (Figure 4c,d). Further analyses examined effector memory (EM), central memory (CM), effector memory T cells that re‐express CD45RA (TEMRA), and naïve CD4⁺ and CD8⁺ T cell subpopulations, revealing distinct basal GSH levels, differential responses to TBHP stimulation, and subpopulation‐specific contributions of GCL and GSR to GSH production (Figure S8, Supporting Information). Neither the expression of GCL nor GSR changed throughout the experiment (Figure S6b, Supporting Information), indicating that TBHP treatment primarily affects the activity of GCL/GSR rather than its expression in this short time course. Similar results were obtained in mouse splenocytes, confirming the applicability of the sensor to study murine cells (Figure S7a, Supporting Information).

To isolate the contributions of GCL and GSR activities in the production of GSH following TBHP treatment, we designed experiments where PBMCs were stimulated with TBHP for 2 h, with or without the addition when the signal stabilized of increasing concentrations of the GCL‐selective inhibitor BSO for another 2 h. The fast reversibility of our sensor enabled us to capture the decrease in intracellular GSH levels due to GCL inhibition, which reached saturation at 2 mm BSO (Figure 4e). We used this saturating concentration to quantify the contributions of GCL and GSR activities (production of GSH in the cell is only due to GCL or GSR) activities based on its impact on the TBHP‐driven GSH increase (Figure 4f,g). Interestingly, GSH levels in CD4^+^ T cells were more dependent on GCL enzymatic activity than on GSR (CD4^+^ T cells: GCL 60% / GSR 40%), whereas CD8^+^ T cells showed an equal dependence on both enzymes for GSH production following TBHP stimulation (CD8^+^ T cells: GCL 50% / GSR 50%) (Figure 4f,g). This finding supports the concept that our approach can effectively capture differences in GCL and GSR activities in T cells.

### 2.6 GCL is Responsible for Producing GSH Following TCR Activation in Human T Cells

**Figure 5:**
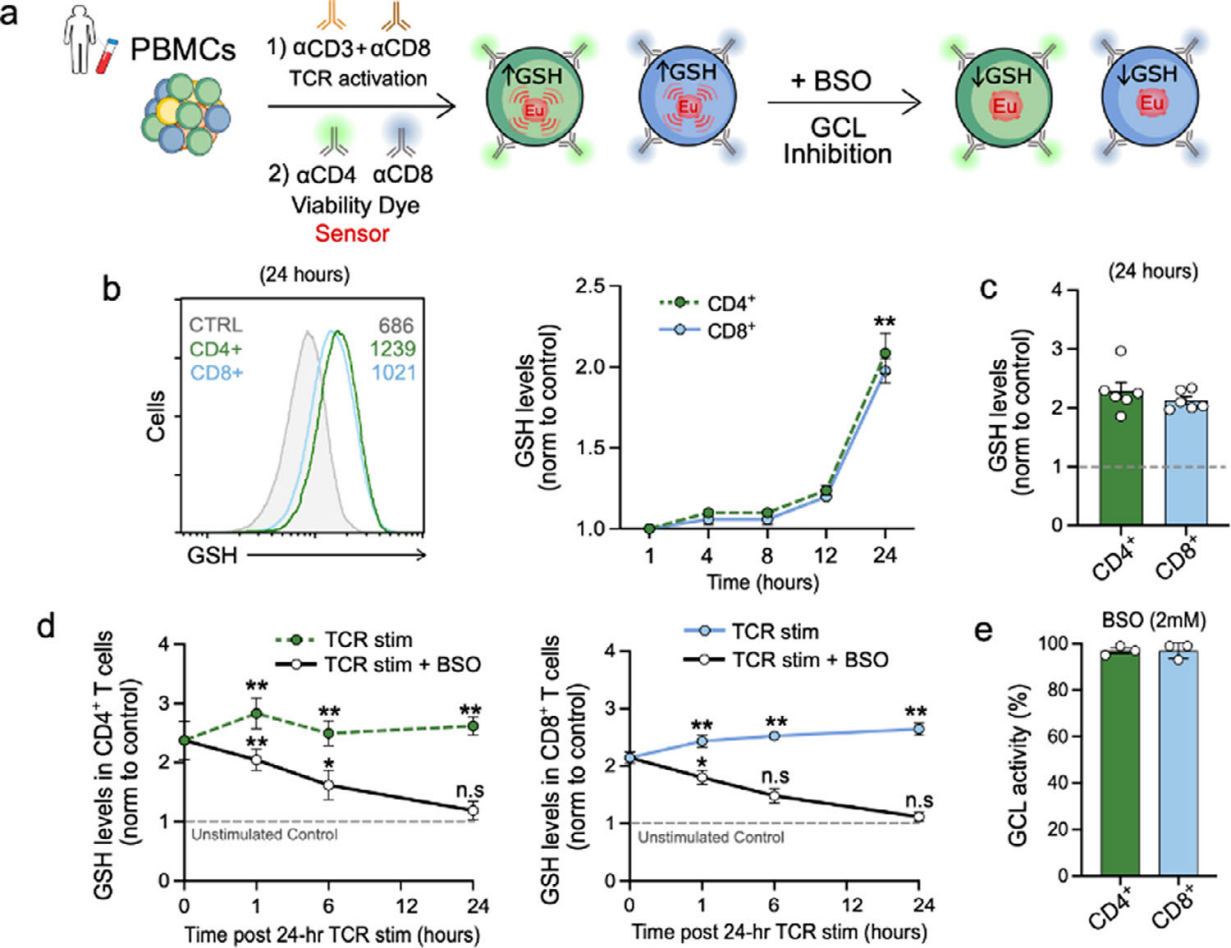
GCL is responsible for producing GSH following TCR activation in human T cells. a) Experimental design diagram. b) Evolution over time of GSH levels measured with **GLed** following TCR activation with aCD3/aCD28 in CD4^+^ (green) and in CD8^+^ (blue) T cells over time (0, 1, 8, and 24 h). c) Representative fluorescent histograms at 24 h after activation, and their summary graph bars (mean ± S.E.M., *n* = 6 per group, three independent experiments, *t*‐test compared to unstimulated control CTRL, ^**^ *p*<0.01). d) Time‐course of GSH levels with or without BSO (2 mm) following TCR activation from CD4^+^ (green) and CD8^+^ (blue), and e) derived average contribution of GCL enzymatic activity to GSH increases (mean ± S.E.M., *n* = 3 per group, three independent experiments, ANOVA followed by Tukey post‐hoc Tukey HSD test compared to levels before BSO addition, ^*^ *p* < 0.05, ^**^ *p* < 0.01).

Taking advantage of **GLed**’s‐based approach to capture dynamic changes in GSH and tease apart the contribution of GCL and GSR enzymatic activities (Figure 4f,g), we aimed to understand how T cells manage GSH homeostasis, and measure the contributions of these two enzymes to GSH production following the activation of primary human T cells, a required step to exert their immune responses (**Figure** **5**a).

We first validated that the sensor captured the generation of GSH elicited by T cell activation. PBMCs were stimulated with anti‐CD3/anti‐CD28 antibodies (TCR activation/co‐stimulation), and GSH levels were measured at different times (1, 4, 8, and 24 h) (Figure 5a). **GLed** reported peak GSH levels at 24 h post‐activation, showing ≈a 2‐fold increase for both CD4^+^ and CD8^+^ T cells (Figure 5b,c). Comparable activation‐induced GSH responses were also observed in mouse splenocytes, further supporting the applicability of the sensor in murine T cell studies (Figure S7b, Supporting Information).

To confirm that **GLed** reflects biologically meaningful levels of GSH in live T cells, we validated its signal against two independent and physiologically relevant indicators of metabolic activity. First, we performed single‐cell analysis of intracellular ROS using the DCFDA probe in CD4⁺ and CD8⁺ T cells after 24 h of TCR stimulation (± BSO). The **GLed** signal positively correlated with ROS levels (Figure S10a, Supporting Information), consistent with GSH synthesis as a compensatory response to oxidative stress. Second, we directly quantified intracellular GSH using a validated colorimetric assay in sorted CD4⁺ and CD8⁺ T cells. As shown in Figure S10b (Supporting Information), GSH levels increased upon activation and were abolished by BSO treatment.

We next assessed the contributions of GCL and GSR to GSH generation following TCR activation/co‐stimulation. As in our TBHP studies, we added BSO at the end of 24 h of activation, when GSH levels were highest (*t* = 0 in Figure 5d). We then compared the evolution of GSH levels over time with a positive control (without BSO) and an unstimulated control (Figure 5d). The GSH levels in samples treated with BSO drastically decreased over time for both CD4^+^ and CD8^+^ T cells, starting at 1 h after BSO treatment (≈30% for both CD4^+^ and CD8^+^ T cells) and eventually reached saturation at levels similar to the control (≈99% for both CD4^+^ and CD8^+^ T cells) (Figure 5d), indicating that the GCL‐mediated pathway was almost the sole contributor for the GSH biosynthesis following activation of CD4^+^ and CD8^+^ (Figure 5e). However, as GCL inhibition was only assessed at a single timepoint (24 h post‐activation), we cannot exclude a potential contribution of GSR during earlier phases of T cell activation.

These findings unveil for the first time the critical role of GCL in GSH production after TCR activation in human T cells and support the concept that measuring the GCL‐driven GSH increase provides a proxy for GLC enzymatic activity in human T cells.

### 2.7 GCL Enzymatic Activity is Correlated With Human T Cell Effector Functions

**Figure 6:**
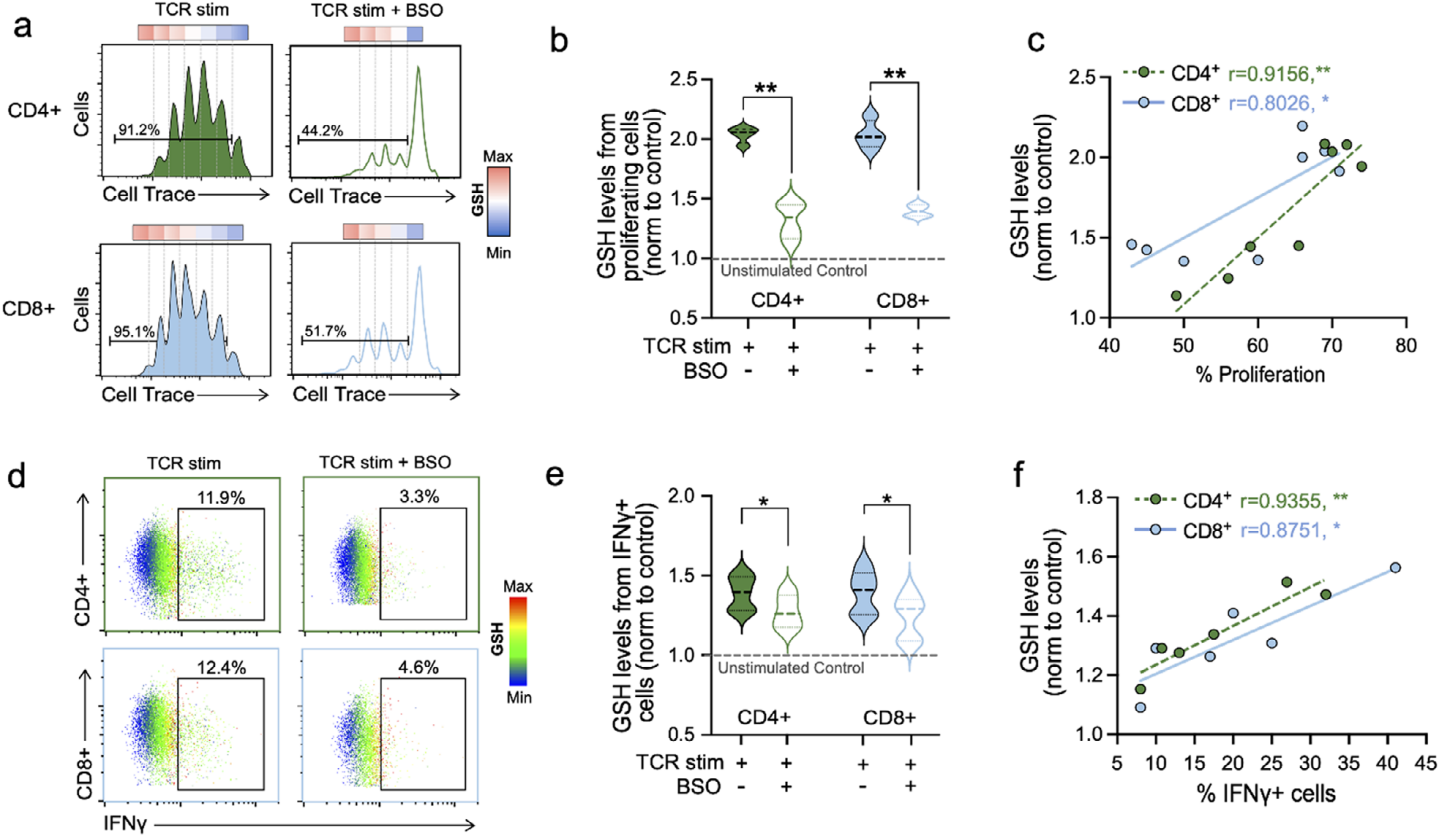
GCL enzymatic activity is correlated with human T cell effector function. a) Representative proliferation histograms from activated (anti‐CD3/anti‐CD28) CD4^+^ T (green), and CD8^+^ T cells (blue) in the absence or presence of BSO (0.5 mm) and GSH levels in each peak of proliferation (heatmaps; blue to red). b) GSH levels in all CD4^+^ and CD8^+^ cells that proliferated in the presence or absence of BSO from (mean ± S.E.M., *n* = 6 per group, three independent experiments, ANOVA with post‐hoc Tukey HSD test, ^**^ *p* < 0.01). c) Correlation analysis between GSH levels and proliferation (%) in all samples (R: correlation coefficient, details on the test). d) Representative scatter plots of IFNγ production in activated T cells overlaid with GSH levels with and without BSO (0.5 mm). e) Summary of the GSH levels in IFNγ^+^ cells (mean ± S.E.M., *n* = 6 per group, three independent experiments, ANOVA with post‐hoc Tukey HSD test, ^*^ *p* < 0.05), and f), correlation analysis between GSH levels and IFNγ^+^ production (%) (R: correlation coefficient, details on the text).

BSO has been shown to inhibit T cell proliferation by blocking the GCL enzyme,^[^ ^59^ ^]^ one of the earliest events following T cell activation, which we confirmed in our experiments (Figure S11, Supporting Information). Based on our results, we hypothesized that the GCL enzymatic activity would reflect the extent of T cell proliferation. To test this, we loaded human primary T cells with a proliferation dye,^[^ ^60^ ^]^ stimulated the cells with anti‐CD3/anti‐CD28 in the presence or absence of a suboptimal dose of BSO, and measured proliferation after 24 h (**Figure** **6**a). This dye labels DNA, and its fluorescence intensity decreases as the cells divide, resulting in distinct peaks where each peak represents a cell division. To study the GCL enzymatic activity in T cell proliferation, we used **GLed** to measure these levels at each peak of proliferation simultaneously (Figure 6a). In the absence of BSO, we observed that the GSH levels increased as the number of divisions increased (higher proliferative peaks), consistent with the idea that proliferation is associated with substantial rewiring of metabolism and thereby GSH homeostasis.^[^ ^61^ ^]^ In the presence of BSO at a lower concentration to capture enough cell divisions (0.5 mm), we observed the same pattern but with overall lower GSH levels in each proliferation peak (Figure 6a,b). Further analysis confirmed that GCL enzymatic activity correlates with the extent of CD4^+^ and CD8^+^ T cell proliferation upon activation (Figure 6c).

One of the most critical effector functions following T cell activation is cytokine production, with IFNγ being a key pro‐inflammatory cytokine primarily released by CD8^+^ and CD4^+^ helper 1 (Th1) effector T cells. We hypothesized that **GLed** could help determine whether IFNγ production induced by T cell activation depends on *de novo* synthesis of GSH. We performed polyclonal stimulation of T cells to induce IFNγ release while simultaneously measuring GCL enzymatic activity in IFNγ^+^  cells with and without BSO treatment. Consistent with our hypothesis, the presence of BSO reduced the number of pro‐inflammatory IFNγ^+^ cells in the culture post‐stimulation (Figure 6d). Interestingly, while BSO did not change GSH levels in IFNγ^−^ cells, IFNγ^+^ cells, which on average showed 1.5 times higher GSH in both CD4^+^ and CD8^+^ T cells, were reduced by ≈20% in the presence of BSO (Figure 6e). An analogous correlation analysis revealed an association between GCL enzymatic activity and IFNγ production (Figure 6f). We also explored the release of additional cytokines such as GZMB and IL‐6, which exhibited similar GSH‐dependent behavior to that observed with IFNγ‐producing cells (Figure S12, Supporting Information). We also evaluated the expression of canonical activation markers (CD25, CD69, and PD‐1) under the same conditions, and observed that CD25 and CD69 were significantly reduced upon BSO treatment, while PD‐1 remained unaffected (Figure S9, Supporting Information), supporting the concept of differential effects of GCL activity on downstream pathways upon T cell activation.

Together, these results provide a previously unrecognized link between GCL activity, cellular GSH, and T cell effector functions, including proliferation and cytokine production, in CD8^+^ and CD4^+^ human primary T cells. They also support GCL enzymatic activity as a potential new measure of T cell activation and function.

### 2.8 Immunosuppressive Drugs Curtail GCL Enzymatic Activity After TCR Activation

Controlling immunosuppressive drug levels is crucial for preventing organ rejection and minimizing toxicity in transplant patients.^[^ ^62^ ^]^ Common methods for measuring these levels include immunoassays such as ELISA, and chromatographic techniques like HPLC and LC‐MS/MS, which provide precise measurements of drug concentrations in the blood.^[^ ^63^ ^]^ While these methods are effective in tracking drug concentrations, they do not directly measure the extent of suppression of T cell function, making optimization of the immunosuppression in each patient extremely challenging.

**Figure 7:**
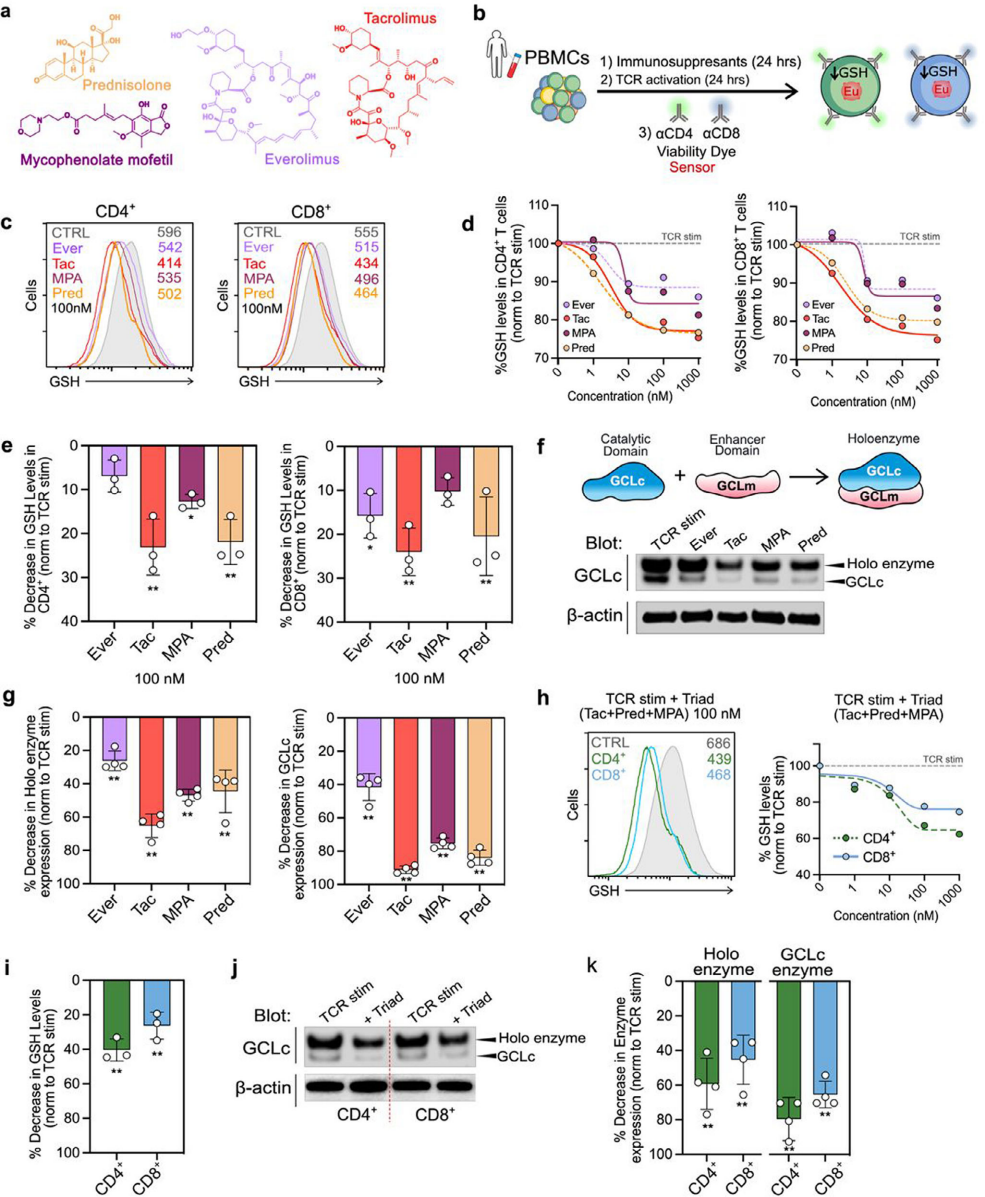
Immunosuppressive drugs curtail GCL‐mediated GSH increase after TCR activation. a) Chemical structure of the four immunosuppressants commonly used in transplant recipients: prednisone (Pred), mycophenolate (MMF), tacrolimus (Tac), and everolimus (Ever). b) Experimental design diagram. c) Representative histograms, and dose‐response‐curves d) of the TCR activation‐induced GSH levels (24 h) in cells pre‐treated for 24 h with Pred, MMF, Ever, and Tac at increasing concentrations (0–1000 nm) from CD4^+^ and CD8^+^ primary human T cells. e) Bar‐graph summary at 100 nm (mean ± S.E.M., *n* = 3 per group, three independent experiments, ANOVA followed by Tukey HSD post‐hoc test, ^*^ *p*<0.05). f) Diagram depicting the GCL subunits and the holoenzyme and representative blot (top), and western blot from culture lysates of TCR‐activated T cells pre‐incubated with vehicle (CTRL) or immunosuppressants (100 nm), and its quantification (ImageJ, see Methods) (mean ± S.E.M., *n* = 4 per group, three independent experiments, ANOVA followed by Tukey HSD post‐hoc test, ^*^ *p*<0.05) g). Lysates were blotted with an antibody recognizing GCLc revealing higher weight band (holoenzyme) and a lower‐weight band (GCLc alone) (lanes were loaded with the same amount of protein). h) Representative histograms, and dose‐response curves in CD4^+^ and CD8+ T cells of the fall in TCR activation‐induced GSH levels (24 h) in cells pre‐treated for 24 h with a triad of tacrolimus, Pred, and MMF at increasing concentrations (0–1000 nm) from CD4^+^ and CD8^+^ primary human T cells. i) Comparison of the decrease in GSH levels after T cell activation with 100 nm of tacrolimus (Tac) or triad treatment in both CD4+ and CD8+ T cells. j) Levels of GCL holoenzyme or GCLc in sorted CD4⁺ and CD8⁺ T cells after pretreatment with triad, and their representative graph bars k).

Given that GCL inhibition produced similar effects (inhibition of T cell effector functions) to those of immunosuppressant drugs commonly used in transplant recipients – prednisone (Pred), mycophenolate (MMF), tacrolimus (Tac), and everolimus (Ever) (**Figure** **7**a) – we hypothesized that changes in GCL enzymatic activity following TCR activation might constitute a proxy for their immunosuppressive effects on T cells. We isolated PBMCs, pre‐incubated them for 24 h with either vehicle, Pred (active form prednisolone), MMF (active form mycophenolic acid, MPA), Tac, or Ever at increasing concentrations (1–1000 nm), and then measured the GSH increase 24 h later before and after TCR activation (Figure 7b,c). While none of the immunosuppressants produced changes in basal GSH levels (Figure S13, Supporting Information), all four drugs inhibited GCL enzymatic activity after TCR activation, with distinct maximum effects and dose dependencies, with Pred, MMF, Ever and Tac having IC_50_s in the nm range (2.41, 7.94, 8.33, and 1.76 nm for CD8^+^ and 1.70, 7.46, 2.84 nm, and 1.91 for CD4^+^, respectively) (Figure 7d). The differences between the drugs were most pronounced at a concentration of 100 nm (Figure 7e).

To verify that these effects were the result of changes in GCL activity, in analogous experiments, we measured through western blot the relative expression of GCLc (catalytic subunit) and its holoenzyme (the most active form of GCL) composed of the GCLc and GLCm and the modifier subunit (see schematic in Figure 7f). Indeed, the reduction of the drop in GSH following activation was associated with a concomitant reduction in the expression of both forms for each drug tested, supporting that these drugs affect GSH homeostasis differently (Figure 7f,g). These differences likely reflect distinct downstream signaling mechanisms. Although both tacrolimus and everolimus bind FKBP12, they inhibit separate pathways: tacrolimus blocks the calcineurin–NFAT axis, whereas everolimus inhibits mTORC1. Given that NFAT and Myc regulate GCLC and GCLM transcription, tacrolimus may more effectively suppress GCL expression and thereby reduce GSH biosynthesis. In contrast, everolimus may allow partial GCL activity through NFAT‐independent or residual Myc‐driven mechanisms. The precise mechanisms by which these drugs modulate GCL remain to be fully elucidated.

Maintenance immunosuppression guidelines for solid organ transplant recipients in many transplant centers include a combination of Pred, MMF, and Tac drugs to minimize rejections.^[^ ^62^, ^64^ ^]^ We decided to test if combined therapy would have additive effects on GCL activity (Figure 7h,i). For the combined therapy, the decrease in TCR activation‐mediated GSH increase in CD4^+^ was markedly higher (≈40% vs ≈20% on average). Interestingly, the effect on CD8^+^ was similar to that of Tac alone (≈20% on average), supporting previous published data describing a lower effect of the combination therapy on CD8^+^ than on CD4^+^ (Figure 7i).^[^ ^65^ ^]^These effects were also reflected in the GCL holoenzyme expression (Figure 7j,k), highlighting the impact of immunosuppressive regimens on glutathione biosynthesis. While these findings demonstrate the robustness of our system to detect immunomodulatory effects in major T cell subsets, future studies will be needed to delineate how GCL and GSR activities vary across more defined subpopulations ‐such as naïve, memory, and effector T cells‐ particularly under TCR stimulation or drug‐induced immunosuppression.

Together, our results reveal, for the first time, a previously unrecognized link between GCL activity, GSH levels, and T cell immunosuppression in the context of immunosuppressive drugs. They also suggest that GCL enzymatic activity can serve as a proxy for assessing human T cell immunosuppression.

## 3 Conclusion

Our findings demonstrate that our **GLed**‐based approach provides unprecedented real‐time insights into GCL activity as a proxy for T cell function and immunosuppression. By enabling precise and reversible measurements of GSH synthesis at the single‐cell level, it has revealed a previously unrecognized link between GCL enzyme activity, GSH homeostasis, and T cell effector functions in human primary T cells. The finding that FDA‐approved immunosuppressive drugs modulate GCL activity, establishes for the first time the clinical relevance of GCL as a potential therapeutic target. Its versatility to be used both with luminescence microscopy and cytometry, highlights the potential of this approach for studying T cell dynamics in clinical samples. These findings open new avenues for monitoring immune status and tailoring immunosuppressive regimens, with broad implications for precision immunotherapy.

## Conflict of Interest

The authors declare no conflict of interest.

## Author Contributions

C.S‐G. and M.N. contributed equally to this work. F.F.G. was responsible for conceptualization, supervision, methodology, visualization, and writing – review and editing. C.S‐G. performed investigation, data analysis, and visualization. M.N. contributed to investigation, data analysis, and visualization. L.E.B. conducted investigation. R.S. contributed to investigation, methodology, and resources. J.M.P. was involved in investigation, data analysis, and methodology. R.H. contributed to supervision, investigation, methodology, and writing – review and editing. A.O. provided supervision, data analysis, visualization, methodology, funding acquisition, and writing – review and editing. M.F. contributed to conceptualization, supervision, methodology, funding acquisition, and writing – review and editing. J.A.G‐V. was responsible for conceptualization, supervision, methodology, funding acquisition, and writing – review and editing.

## Supporting information



Supporting Information

## Data Availability

The data that support the findings of this study are available from the corresponding author upon reasonable request.
