# Corrigendum: Gamma‐Glutamyl Cysteine Ligase Activity as a Proxy for Human T Cell Function and Drug‐Induced Immunosuppression

**DOI:** 10.1002/advs.202520906

**Published:** 2025-12-01

**Authors:** Francisco Fueyo‐González, Carmen Salto‐Giron, Mehek Ningoo, Laura Espinar‐Barranco, Rafael Salto, Jose Manuel Paredes, Rosario Herranz, Angel Orte, Miguel Fribourg, Juan A. González‐Vera

**Affiliations:** ^1^ Instituto de Química Médica (IQM‐CSIC) Juan de la Cierva 3 Madrid 28006 Spain; ^2^ Department of Pharmacological Sciences Center of Translational Medicine and Pharmacology Icahn School of Medicine at Mount Sinai New York NY 10029 USA; ^3^ Department of Medicine Translational Transplant Research Center Immunology Institute Icahn School of Medicine at Mount Sinai New York NY 10029 USA; ^4^ Nanoscopy‐UGR Laboratory Departamento de Fisicoquímica Unidad de Excelencia de Química Aplicada a Biomedicina y Medioambiente Facultad de Farmacia Universidad de Granada Campus Cartuja Granada 18071 Spain; ^5^ Departamento de Bioquímica y Biología Molecular II Unidad de Excelencia de Química Aplicada a Biomedicina y Medioambiente Facultad de Farmacia Universidad de Granada Campus Cartuja Granada 18071 Spain

https://doi.org/10.1002/advs.202501179

Corrigendum

This corrigendum provides a correction to the acknowledgements section. The corrected version is given below.

