# Supplementary material for: Gamma‐Glutamyl Cysteine Ligase Activity as a Proxy for Human T Cell Function and Drug‐Induced Immunosuppression
Source: Adv Sci (Weinh). 2025 Jun 30;12(37):e01179. doi: 10.1002/advs.202501179 (PMC12499489; doi:10.1002/advs.202501179)
Supplement: Supplementary file 1 — Supporting Information [file ADVS-12-e01179-s001.docx]

Supporting Information

**Gamma-Glutamyl Cysteine Ligase Activity as a Proxy for Human T Cell Function and Drug-Induced Immunosuppression**

Francisco Fueyo-González^1,2,3,^*, Carmen Salto-Giron^4,⧫^, Mehek Ningoo^3,⧫^, Laura Espinar-Barranco^3,4^, Rafael Salto^5^, Jose Manuel Paredes^4^, Rosario Herranz^1^, Angel Orte^4^, Miguel Fribourg^3,^* and Juan A. González-Vera^4,^*

*^1^ Instituto de Química Médica (IQM-CSIC), Juan de la Cierva 3, 28006 Madrid, Spain*

*^2^ Department of Pharmacological Sciences, Center of Translational Medicine and Pharmacology, Icahn School of Medicine at Mount Sinai, New York, USA*

*^3^ Department of Medicine, Translational Transplant Research Center, Immunology Institute, Icahn School of Medicine at Mount Sinai, New York, USA*

*^4^ Nanoscopy-UGR Laboratory. Departamento de Fisicoquímica, Unidad de Excelencia de Química Aplicada a Biomedicina y Medioambiente, Facultad de Farmacia, Universidad de Granada, Campus Cartuja, 18071, Granada, Spain*

*^5^ Departamento de Bioquímica y Biología Molecular II, Unidad de Excelencia de Química Aplicada a Biomedicina y Medioambiente, Facultad de Farmacia, Universidad de Granada, Campus Cartuja, 18071, Granada, Spain*

**e-mail:* [*gonzalezvera@ugr.es*](mailto:gonzalezvera@ugr.es)*;* [*miguel.fribourg@mssm.edu*](mailto:miguel.fribourg@mssm.edu)*;* [*francisco.fueyogonzalez@mssm.edu*](mailto:francisco.fueyogonzalez@mssm.edu)

^⧫^These authors contributed equally

**Table of Contents PAGE**

**I. General methods** S2

**II. General Reagents and Materials** S2

**III. Experimental synthetic methods** S4

**IV. Luminescence experiments** S6

**V. Luminescence microscopy**  S9

**VI. Human subjects** S9

**VII. T cell viability** S10

**VIII. T cell stimulation** S11

**IX. GCL staining** S13

**X. Proliferation assay** S13

**XI. Cytokine release** S14

**XII. Flow cytometry** S15

**XIII. Western Blot and Immunoprecipitation** S15

**XIV. GSH levels in the presence of immunosuppressive drugs** S15

**XV. NMR spectra (^1^H-NMR and ^13^C-NMR) of 3 and 4** S16

**XVI. References** S19

## **I. General Methods**

All reagents were of commercial quality. Solvents were dried and purified by standard methods. Analytical TLC was performed on aluminum sheets coated with a 0.2 mm layer of silica gel 60 F_254_. Silica gel 60 (230-400 mesh) was used for flash chromatography.

HPLC-MS was performed on a Sunfire C_18_ (4.6×50 mm, 3.5 μm) column at 30°C, with a flow rate of 1 mL/min and gradient of 0.1% of formic acid in CH_3_CN (solvent A) in 0.1% of formic acid in H_2_O (solvent B) was used as mobile phase. Electrospray in positive mode was used for ionization. NMR spectra were recorded using Bruker 300 spectrometer (GmbH). The NMR spectra assignments were based on COSY, HSQC, and HMBC spectra. High resolution mass spectra (HRMS) were recorded on an Agilent 6520 Q-TOF instrument with an ESI source. UV-visible spectroscopy measurements were made at 25 Cº on a Lambda 35, Perkin Elmer, UV-vis spectrophotometer. Steady-state fluorescence emission spectra were obtained on a Jasco FP-8300 Spectrofluorometer (Jasco, Tokyo, Japan). Lifetime experiments were acquired in a Varian Cary Eclipse Spectrofluorometer. Starna and Hellma quartz cuvettes of 1 cm path length and several volumes were employed. Time-resolved luminescence emission spectra were obtained on a CLARIOstar® spectrofluorimeter (BMG LABTECH). Plate: Greiner assay plate (greiner bio-one), 96-well, no lid, flat bottom, medium binding surface, non-sterile, black polystyrene.

**II. General Reagents and Materials**

**Reagents and mouse strains**

| REAGENT or RESOURCE | SOURCE | IDENTIFIER |
| --- | --- | --- |
| Antibodies |  |  |
| BV450 anti-human Foxp3 | eBiosciences | Cat# 48-4776-42 RRID:AB_1834364 |
| αCD3 human | BD Biosciences | Cat# 566685 |
| FITC anti-human Foxp3 | eBiosciences | Cat# 11-4777-42 RRID:AB_1518812 |
| PerCP Cy5.5 anti-human CD4 antibody | BD Biosciences | Cat# 552838 RRID:AB_394488 |
| FITC anti-human CD4 antibody | eBiosciences | Cat# 11-0042-86 RRID:AB_464898 |
| Chemical, Peptides and Recombinant Proteins |  |  |
| Recombinant TGFβ1 | Peprotech | Cat# 100-21C |
| Recombinant human IL-2 | BD Pharmingen | Cat# 554603 |
| CellTrace violet Cell Proliferation | Thermofisher | Cat# C34557 |
| eFluor 780 Fixable viability dye | eBioscience | Cat# 65-0865-14 |
| ACK lysis buffer | Roche | Cat# 11814389001 |
| 5X RIPA Buffer | ThermoScientific | Cat# J62524.AE |
| Pierce BCA protein assay | ThermoScientific | Cat# 23228 |
| Protein G MagBeads | GenScript | Cat# L00274 |
| Laemmli SDS sample Buffer, reducing (6x) | Millipore | Cat# J61337.AC |
| Immobilon®- P Transfer Membrane (Pore 0.45μm) | Millipore | Cat# IPVH00010 |
| Immobilon® Western Chemiluminescent HRP Substrate | ThermoScientific | Cat# WBKLS0100 |
| TritonX-100 | Immunechem | Cat# A16046.AE |
| AffinityScript MultiTemp RT | Agilent | Cat# 600105 |
| Cell-permeant ROS-sensitive dye DCFDA | Sigma-Aldrich | Cat# 287810 |
| Dulbecco’s modified Eagle’s medium (DMEM) | Cultek | Cat# SH30565.01 |
| Fetal bovine serum (FBS) | Cultek | Cat# S1400-500 |
| Glutamine | Cultek | Cat# SV30160.03 |
| Penicillin- Streptomycin | SigmaAldrich | Cat# P4458-100ML |
| HEPES buffer | SigmaAldrich | Cat# H0887-100ML |
| NaCl | SigmaAldrich | Cat# S3014-5KG |
| Critical Commercial Assays |  |  |
| EasySep™ Human Naïve CD4+ T Cell Isolation Kit | STEMCELL Technologies | Cat# 19555 |
| aCD3/aCD28 stimulating beads human | Gibco | Cat# 11-456D |
| Intracellular/transcription factor staining buffer kit | eBiosciences | Cat# 00-5523-00 |
| Glutathione Colorimetric Detection Kit | Themorfisher | Cat# EIAGSHC |
| Experimental Models: Organisms/Strains |  |  |
| Hepatocarcinoma HepG2 cells | Cell Culture Facility (University of Granada, Spain) | ECACC: 85011430 |

## **III. Experimental Synthetic Methods**

**Scheme S1.** Synthesis of the DO3A chelates-based lanthanide GSH sensors **5** (**GLed**, Ln = Eu^3+^), **6** (Ln = Tb^3+^), **7** (Ln = Sm^3+^) and **8** (Ln = Dy^3+^).

**Synthesis of tri-*tert*-butyl 2,2',2''-(10-(2-(8-methoxy-2-oxo-1,2-dihydrocyclopenta[*de*]quinoline-3-carboxamido)ethyl)-1,4,7,10-tetraazacyclododecane-1,4,7-triyl)triacetate (3)**. HBTU (130 mg, 0,35 mmol), HOBt (53 mg, 0,35 mmol) and DIPEA (60 µL, 0,35 mmol) were added to a solution of 8-methoxy-2-oxo-1,2-dihydrocyclopenta[*de*]quinoline-3-carboxylic acid (**1**)^1^ (70 mg, 0,29 mmol) and DO3A^t^Bu-NEtNH_2_ (**2**)^2^ (176 mg, 0,31 mmol) in dry DMF (10 mL). The mixture was stirred under argon atmosphere for 16 h. Then, the mixture was evaporated to dryness and the residue was purified by flash chromatography, using 0-5% gradient of MeOH in CH_2_Cl_2_ as eluent to give the desired amide **3** as an orange syrup (120 mg, 53 %). HPLC-MS (5-95% gradient of solution A in B, 10 min) *t*_R_ = 7,32 min. ^1^H-RMN [(CD_3_)_2_CO, 400 MHz] δ: 1.44 and 1.52 (2s, 27 H), 2.36-2.88 (m, 16 H), 3.13-3.54 (m, 6 H), 3.61 (m, 4 H), 3.99 (s, 3H), 6.95 (d, *J* = 7.5 Hz, 1 H), 7.20 (d, *J* = 7.5 Hz, 1 H), 7.24 (d, *J* = 5.5 Hz, 1 H), 7.68 (d, *J* = 5.5 Hz, 1 H). 10.16 (t. 1H. *J* = 6 Hz,).^13^C-RMN [(CD_3_)_2_CO, 100 MHz] δ: 26.1, 35.3, 42.0, 54.0, 55.4, 55.5, 55.8, 81.3, 81.4, 110.8, 118.3, 118.4, 119.8, 124.3, 127.9, 131.0, 140.0, 147.4, 154.0, 163.5, 163.6, 163.7 and 172.6. HRMS (ESI): Calcd. for C_41_H_62_N_6_O_9_ ([M+H]+): 783.4578, Found: 783.4721.

**Synthesis of 2,2',2''-(10-(2-(8-methoxy-2-oxo-1,2-dihydrocyclopenta[*de*]quinoline-3-carboxamido)ethyl)-1,4,7,10-tetraazacyclododecane-1,4,7-triyl)triacetic acid (4)**. A solution of the tri-*ter*-butyl ester 3 (40 mg, 0.05 mmol) in TFA (2mL) was stirred at room temperature under argon atmosphere for 1.5 h. Then, the mixture was evaporated to dryness and the residue was purified by semipreparative HPLC in a SunFire Prep C_18_ OBD de 5 µm (19x150 mm) column, obtaining the desired free acid **4** (8 mg, 25 %) as an orange solid, which was lyophilized. HPLC-MS (2-30% gradient of solution A in B, 10 min) *t*_R_ = 8,80 min. ^1^H-NMR (D_2_O, 500 MHz) δ: 2.50-3.54 (m, 22 H), 3.63 (s, 3H), 3.68 (m, 4H), 6.33 (d, *J* = 7 Hz, 1 H), 6.60 (d, *J* = 5 Hz, 1 H), 6.64 (d, *J* = 7 Hz, 1 H), 6.73 (d, *J* = 5 Hz, 1 H), 8.24 (s, 1H). ^13^C-RMN D_2_O, 125 MHz] δ: 35.2, 48.4, 50.4, 51.1, 54.2, 56.0, 56.4, 111.0, 115.9, 117.6, 121.7, 123.1, 126.1, 130.1, 141.3, 147.6, 153.1, 163.4, 165.6, 169.8. HRMS (ESI): Calcd. for C_29_H_38_N_6_O_9_ ([M+H]^+^): 615.2700, Found: 615.2791.

**Synthesis of lanthanide-based 2,2',2''-(10-(2-(8-methoxy-2-oxo-1,2-dihydrocyclopenta-[*de*]quinoline-3-carboxamido)ethyl)-1,4,7,10-tetraazacyclododecane-1,4,7-triyl)triacetates 5** (**GLed**, Ln = Eu^3+^), **6** (Ln = Tb^3+^), **7** (Ln = Sm^3+^) and **8** (Ln = Dy^3+^). A 50 mM solution of the corresponding lanthanide trichloride salt (EuCl_3_, TbCl_3_, SmCl_3_ or DyCl_3_) in HCl 1 mM (250 µL) was added to a 10 mM solution of the triacid **4** in HEPES buffer 50 mM, pH 7.4 (250 µL) and the mixture was stirred at room temperature for 16 h. Finally, the desired DO3A chelates **5**-**8** were purified on NAP-5 columns (GE Healthcare). HRMS (ESI): Calcd. for **5** (**GLed**, Ln = Eu^3+^) C_29_H_35_EuN_6_O_9_ ([M+H]^+^): 765.1678, Found: 765.1810. Calcd. for **6** (Ln = Tb^3+^) C_29_H_35_N_6_O_9_Tb ([M+H]^+^): 771.1719, Found: 771.1846. Calcd. for **7** (Ln = Sm^3+^) C_29_H_35_N_6_O_9_Sm ([M+H]^+^): 764.1663, Found: 764.1506. Calcd. for **8** (Ln = Dy^3+^) C_29_H_35_DyN_6_O_9_ ([M+H]^+^): 775.1320, Found: 775.1573.

## **IV. Luminescence experiments**

Time-gated (TG) emission spectra were made with a CLARIOstar® spectrofluorimeter. The spectra were recorded between 462 and 800 nm, and all measurements were made using the following settings: excitation wavelength 337 nm (EX TR excitation filter); delay time 0.4 ms; stepwidth 1.0 nm; emission bandwidth 10 nm; dichroic filter LP TR; gain: 1800; focal height 8 nm; 20 flashes per well, target value 10%. All the spectra were corrected for background luminescence by subtracting a blank scan of the solvent solution. Experiments were performed in triplicate or quadruplicate. Error values indicate standard deviation from average.

***a. Lanthanide luminescence sensitization of DO3A chelates-based GSH sensors***

The ability of chelates **5** (**GLed**, Ln = Eu^3+^), **6** (Ln = Tb^3+^), **7** (Ln = Sm^3+^) and **8** (Ln = Dy^3+^) (5 μM) to serve as effective Michael acceptors for GSH, sensitizing the luminescence of lanthanide ions, was spectroscopically analyzed upon reaction with GSH (2000 equivalents) in HEPES buffer at pH 7.4 for 3 hours (Fig. S1). The oxidized compounds **5**-**8** practically did not sensitize the emission of the corresponding lanthanide ions, yielding negligible luminescence. However, upon reaction with GSH (λ_ex_ = 337 nm), **5(GLed)**-GSH clearly sensitized the luminescence of Eu^3+^, leading to the appearance of the ^5^D_0_ →^7^F_2_ and ^5^D_0_ →^7^F_4_ bands at 615 nm and 695 nm, respectively. Under the same conditions, **6**-GSH sensitized the luminescence of the ^5^D_4_→^7^F_6_ (490 nm) and ^5^D_4_→^7^F_5_ (540-550 nm) Tb^3^ bands, but to a much lower extent than **5(GLed)**-GSH (5.7 times lower), while Sm^3+^ and Dy^3+^ chelates (**7**-GSH and **8**-GSH) exhibited negligible luminescence emission. Remarkably, upon reaction with GSH, **5(GLed)**-GSH led to a significant luminescent increase of 26.5-fold of the Eu^3+^ emission, while **6**-GSH showed a 2.5-fold increase. These results moved us to focus on the Eu^3+^-based GSH detection sensor **GLed**.





**Figure S1.** Representative TG emission spectra (λ_ex_ = 337 nm, n=4) of chelates **5** (**GLed**, Ln = Eu^3+^), **6** (Ln = Tb^3+^), **7** (Ln = Sm^3+^) and **8** (Ln = Dy^3+^) (5 μM) before and after reaction with GSH (2000 equivalents) in HEPES buffer at pH 7.4 for 3 hours.

***b. Fluorescence lifetimes determination***

Lifetime experiments of **5** (**GLed**) and the lanthanide complexes of the addition products of GSH, Hcy, and Cys to **GLed** (**5(GLed)**-GSH, **5(GLed)**-Hcy and **5(GLed)**-Cys) were acquired in a Varian Cary Eclipse Spectrofluorometer at room temperature using the following conditions: excitation wavelength 320 nm; emission wavelength 616 nm; excitation slit width 5.0 nm, emission slit width 5.0 nm; total decay time 15.0 ms; delay time 0.1 ms; gate time 0.2 ms; number of cycles 20; PMT detector voltage 600 V.

***c. Luminescence of GLed in the presence of other potential interfering species***





**Figure S2.** Fluorescence emission intensity (λ_ex_ = 337 nm) of **GLed** at 616 nm (5 μM in HEPES buffer 50 mM, pH 7.4) after addition of 100 equivalents of GSH and the possible interferents Hcy, Cys, SH_2_, Fe^2+^, Na_2_S_2_O_3_, HClO and NaNO_2_.

***d. Quantitative power of GLed for physiologically relevant GSH determinations***


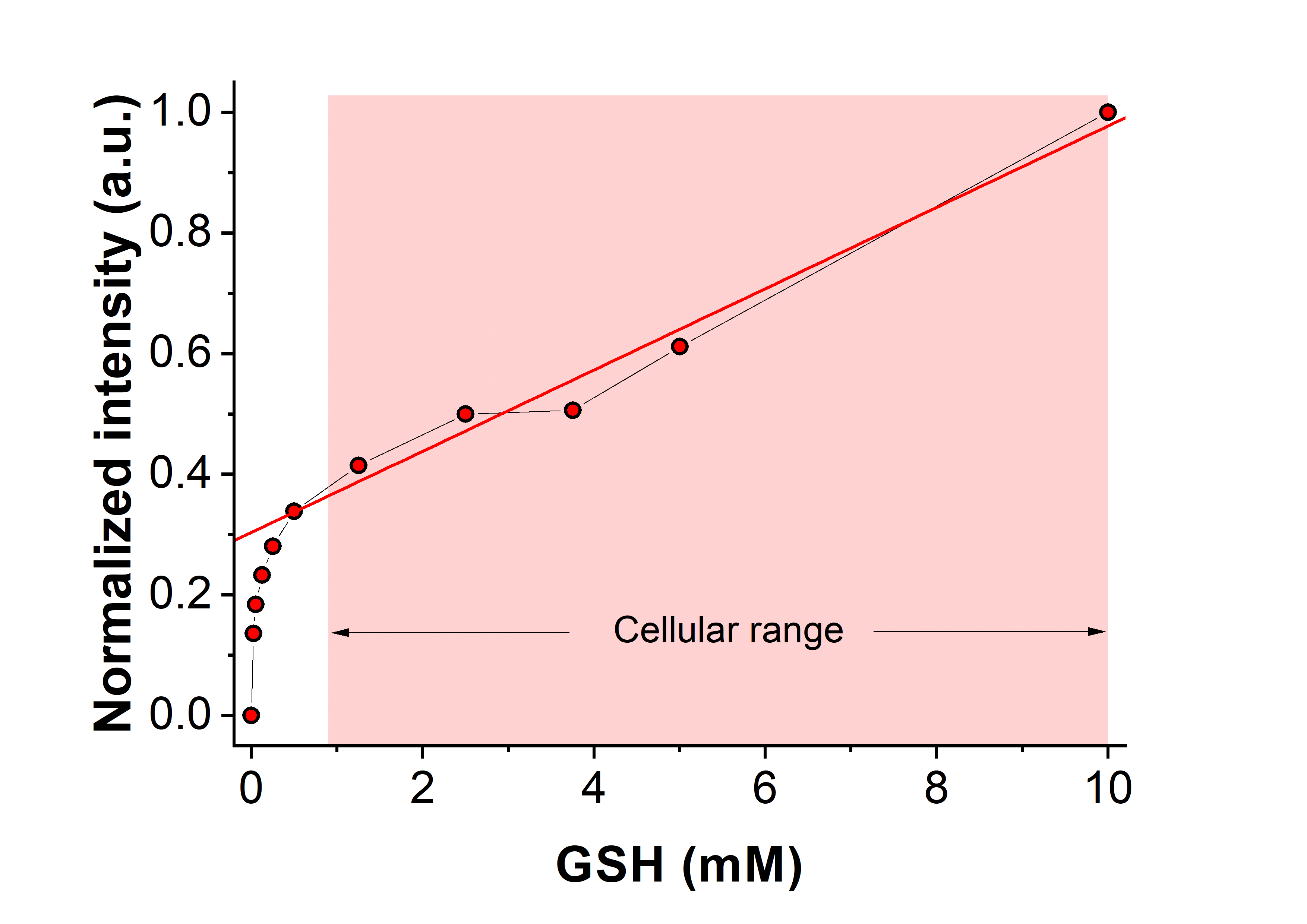


**Figure S3.** The Eu^3+^ luminescence of **GLed** (λ_ex_ = 337 nm) shows a linear response to increasing GSH concentrations within the physiological range of 1-10 mM and exhibits a highly sensitive response to sub-mM levels of GSH.

***e. Dilution experiments to demonstrate the immediate reversibility of GLed***


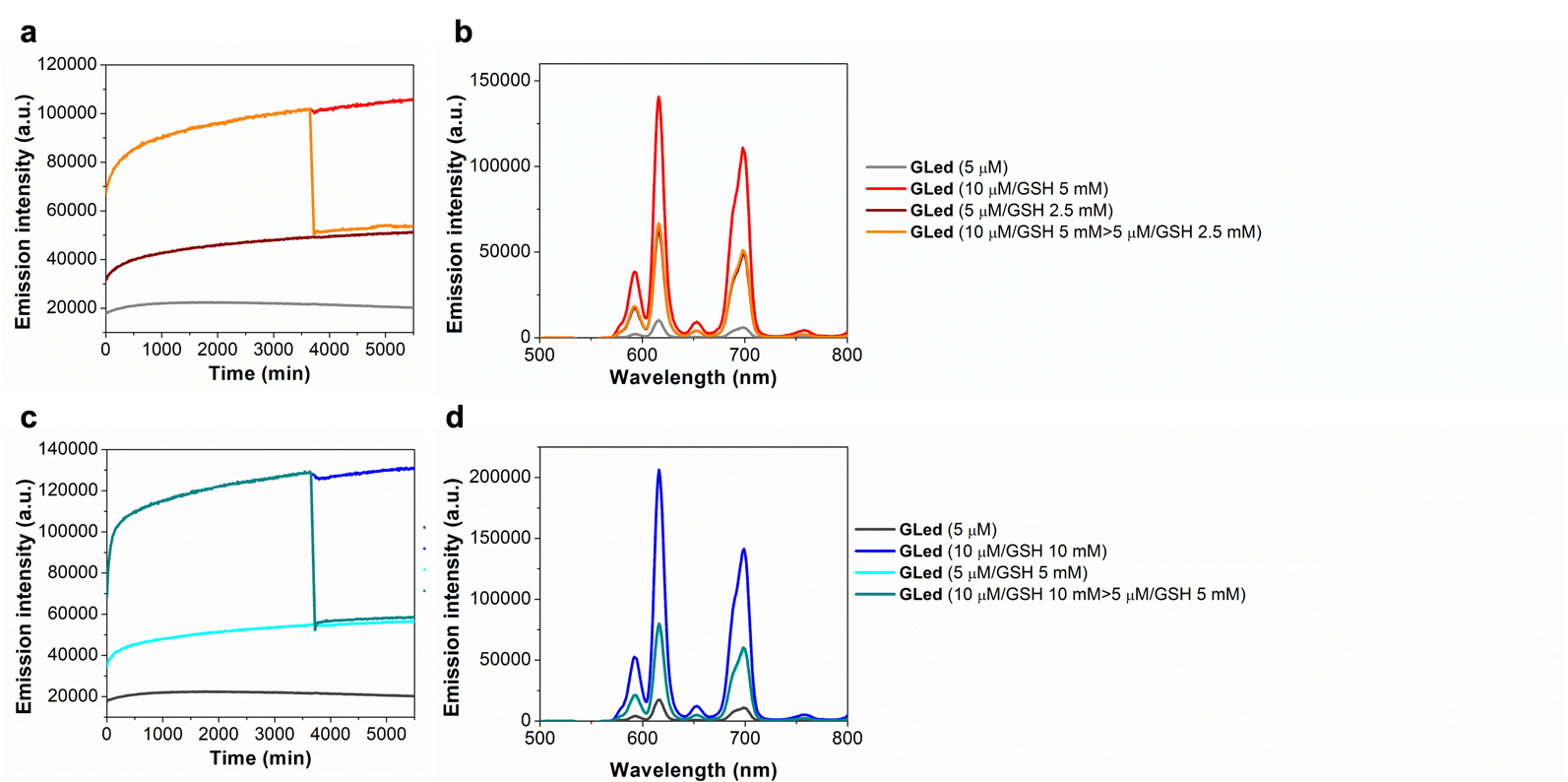


####

**Figure S4.** **a** Changes in the Eu^3+^ luminescence emission intensity over time at 616 nm (λ_ex_ = 337 nm), and **b** TG emission spectra (λ_ex_ = 337 nm) of a solution **GLed** (10 μM) and GSH (5 mM) in HEPES buffer at pH 7.4, diluted two-fold, resulting in intensity values comparable to those obtained from a **GLed** (5 μM) solution upon the addition of GSH (2.5 mM). **c** Changes in the Eu^3+^ luminescence emission intensity over time at 616 nm (λ_ex_ = 337 nm), and **d** TG emission spectra (λ_ex_ = 337 nm) of a solution **GLed** (10 μM) and GSH (10 mM) in HEPES buffer at pH 7.4, diluted two-fold, resulting in intensity values comparable to those obtained from a **GLed** (5 μM) solution upon the addition of GSH (5 mM).

## **V. Luminescene microscopy**

Hepatocarcinoma HepG2 cells were supplied by the Cell Culture Facility (University of Granada, Spain). Cells were grown at 37 °C in Dulbecco’s modified Eagle’s medium (DMEM) supplemented with 10% (v/v) fetal bovine serum (FBS), 2 mM glutamine, 100 U/mL penicillin, and 0.1 mg/mL streptomycin. For experiments, cells were seeded onto a 8-well iBIDI microscopy plate at a density of 1.6×10^5^ cells/well for 24 h to reach a cell confluence of 80%. For imaging, the cell culture medium was first removed from each well and cells were washed twice with HEPES 50 mM pH 7.4, NaCl 0.9%. Subsequently, **GLed** (5 μM) was added and incubated for 1 hour in Hepes (50 mM, pH 7.4). HepG2 cells were pretreated with *N*-ethylmaleimide (NEM, 500 μM) to eliminate the endogenous GSH (only for the 0-14 mM GSH addition experiment, Fig. 3 in main text).

Confocal and time-gated (TG) luminescence microscopy experiments were carried out on an Abberior Expert Line microscope (Abberior Instruments GmbH, Germany). The excitation source was a 375-nm pulsed laser, working at a repetition rate of 40 MHz. The detection of the Eu^3+^ emission was performed on a hybrid after a bandpass filter with the 580–630 nm spectral range. For conventional confocal imaging, the size (area) of the raw images obtained ranged from 40×40 µm^2^ to 80×80 µm^2^, using a pixel resolution of 500 to 1000 nm/pixel, and a dwell time (time per pixel) of 50 μs. For TG imaging microscopy, the same excitation source was employed but programming a train of pulses of 240 μs, to improve the excitation rate of long-lived lanthanide complexes,^3^ followed by an acquisition time window in which the laser was turned off, for a total dwell time (time per pixel) of 6.012 ms. The area of the images also ranged from 40×40 µm^2^ to 80×80 µm^2^, but using a lower pixel resolution of 200 nm/pixel due to the longer acquisition times required.

For the analysis of the **Gled** images, we employed Fiji (a distribution of ImageJ)^4^. The average intensity per pixel in the Eu^3+^ channel was quantified using an automatic threshold criterion (“Li”) to the region of interest (ROI) of the imaged cells. After quantification, a spatial binning (4x4 pixels), a Gaussian filter (1.5 pixels) and pseudocolor scale (LUTs) were set for better image representation.

**VI. Human subjects**

For the experiments conducted in human primary cells, we utilized peripheral blood mononuclear cells (PBMCs) derived from buffy coats provided by anonymous donors to the New York Blood Bank. The sex and age of the donors (all over 18 years old) were unknown to the researchers. The Icahn School of Medicine's Institutional Review Board (IRB) classified this study as not involving Human Subject Research. The number of donors for each experiment is noted in the figure legends, and individual data points for each donor are represented in the bar graphs.

**VII. T cell viability**

***a. Hepatocarcinoma*** ***HepG2 cells***

20000 cells/well were seeded in 48-well plates and incubated with 0-50 μM of **GLed**. Cytotoxicity was evaluated after 24 h of incubation using the 3-(4,5-dimethylthiazol-2-yl)-2,5-diphenyl-2H-tetrazolium bromide (MTT) method (n=4).^5^ Results are reported as the percentage of cell viability based on the untreated control cells normalized to 100%.


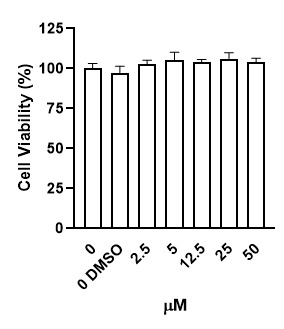


**Fig. S5.** Cytotoxicity study of **GLed** (0-50 μM) after 24 h of incubation (n=4).

***b. Viability of*** ***PBMCs***

PBMCs were incubated with the GLed sensor at concentrations ranging from 0 to 50 μM in complete RPMI medium for 24 hours. After incubation, cells were washed and stained for 30 minutes at 4°C in PBS or staining buffer (2% rat serum, 2 mM EDTA) using fluorescently conjugated anti-CD4 and anti-CD8 antibodies, along with a fixable viability dye (eFluor™ series, Invitrogen) added at this stage to assess cell viability.

For experiments involving oxidative stress (TBHP or TBHP + BSO), or TCR stimulation (using αCD3/αCD28 beads), the viability dye was added after treatment, immediately before flow cytometry acquisition. This allowed detection of potential treatment-induced cytotoxic effects based on membrane integrity.


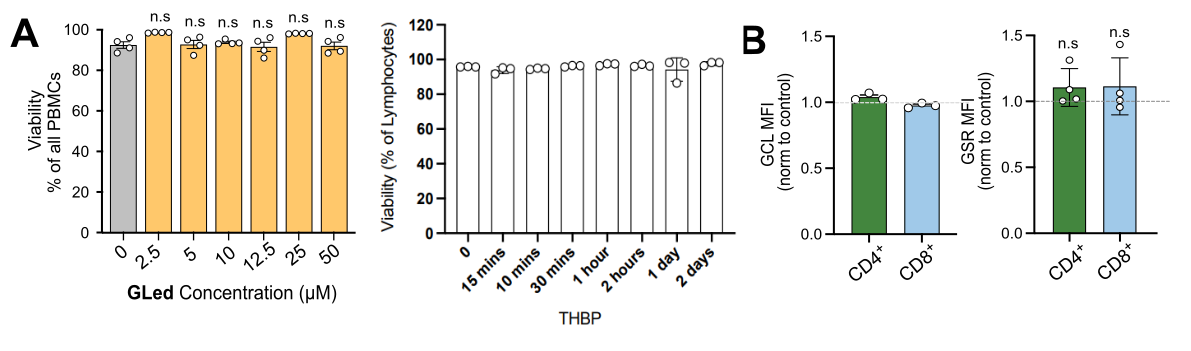


**Fig. S6. a (left)** % Viability of PBMCs following incubation with **GLed** for 24 hours at increasing concentrations (0-50 μM) (mean ± S.E.M., n=4 per group, three independent experiments, ANOVA with Tukey HSD, *p<0.05) or after TBHP treatment (mean ± S.E.M., n=4 per group, three independent experiments, ANOVA with Tukey HSD, *p<0.05) **(right)**. **b** **(left)** GCL expression after stimulation with TBHP for 12 hours (mean ± S.E.M., n=3 per group, three independent experiments, t-test compared to control). **(right)** GSR expression after stimulation with TBHP for 2 hours (mean ± S.E.M., n=4, t-test compared to control).

**VIII. T cell stimulation**

***a. TBHP and TCR stimulation***

PBMCs or splenocytes were stimulated with TBHP (3.5 μM) or αCD3/αCD28 (TCR activation/co-stimulation antibodies). For TBHP experiments, cells were stained with fluorescently conjugated antibodies (CD4, CD8, CD45RA, CD27, CD25, CD127, PD-1, CD69), viability dye and **GLed** in PBS or a buffer consisting of 2% rat serum 2 mM EDTA for 45 min. Cells were then washed and stimulated with TBHP at different times (0-12 hours) in cRPMI. Cells were washed, resuspended in PBS and assessed by flow cytometry.

For αCD3/αCD28 experiments, cells were stimulated at different times (0-24 hours) and then washed and stained with fluorescently conjugated antibodies (CD4 and CD8), viability dye and **GLed** in PBS or a buffer consisting of 2% rat serum 2 mM EDTA. Afterwards, cells were washed, resuspended in PBS assessed by flow cytometry.


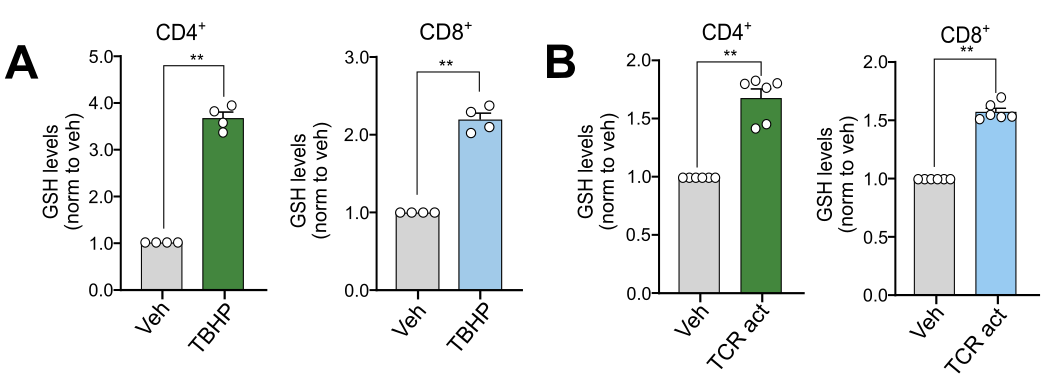


**Fig. S7. a** GSH levels (MFI) in mouse CD4 and CD8 T cells, normalized to vehicle after stimulation with TBHP for 2 hours (mean ± S.E.M., n = 4 mice per group; three independent experiments; t-test, *p < 0.05).**b** GSH levels (MFI) in mouse CD4 and CD8 T cells, normalized to vehicle after stimulation with anti-CD3/CD28 for 24 hours (mean ± S.E.M., n = 6 mice per group; three independent experiments; t-test, *p < 0.05).


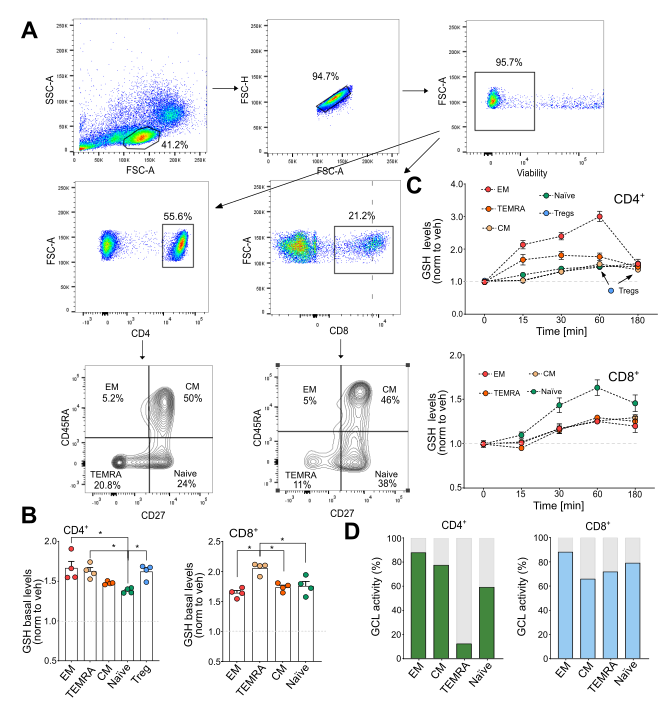


**Fig. S8. a** Representative scatter plots showing the distribution of T cell subsets: effector memory (EM), central memory (CM), TEMRA, and naïve. **b** Basal GSH levels (MFI) in the above subpopulations of CD4⁺ and CD8⁺ T cells from PBMCs, normalized to vehicle. **c** GSH levels in these subpopulations after stimulation with TBHP for 180 min (mean ± S.E.M., n = 3 per group; three independent experiments). **d** Bar graph summarizing the average GCL activity (%) in the same subpopulations following 2-hour treatment with TBHP and BSO (2 mM) (mean ± S.E.M., n = 3 per group; two independent experiments; t-test, p < 0.01).


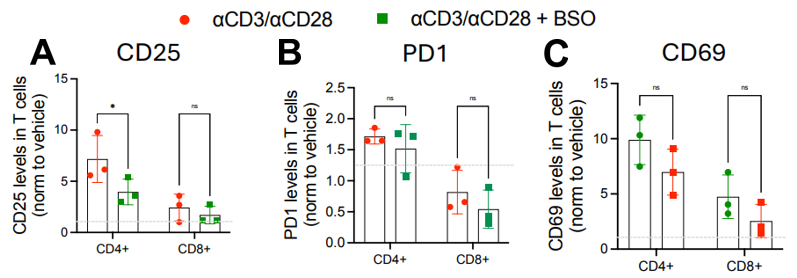


**Fig. S9.** **a** Bar graph comparing CD25 expression levels in CD4⁺ and CD8⁺ T cells after 24 hours of stimulation with anti-CD3/CD28 antibodies ± BSO (2 mM). **b** Same as (a), showing PD-1 expression levels.

**c** Same as (a), showing CD69 expression levels (mean ± S.E.M., n=3 per group, three independent experiments, t-test, *p<0.05).

***b. ROS and GSH quantification***

PBMCs were stimulated for 24 hours with anti-CD3/anti-CD28 antibodies to mimic TCR activation and co-stimulation. For ROS analysis, cells were stained with fluorescently conjugated antibodies (CD4, CD8) and incubated with the cell-permeant ROS-sensitive dye DCFDA (Sigma-Aldrich). ROS levels were measured at the single-cell level by flow cytometry. In selected experiments, cells were co-treated with the GCL inhibitor BSO (2 mM) to evaluate the contribution of de novo GSH synthesis to redox homeostasis.

For total GSH quantification, CD4⁺ and CD8⁺ T cells were sorted following TCR stimulation (±BSO) and analyzed using the Glutathione Colorimetric Detection Kit (Themorfisher), a luminescence-based method for intracellular GSH detection.


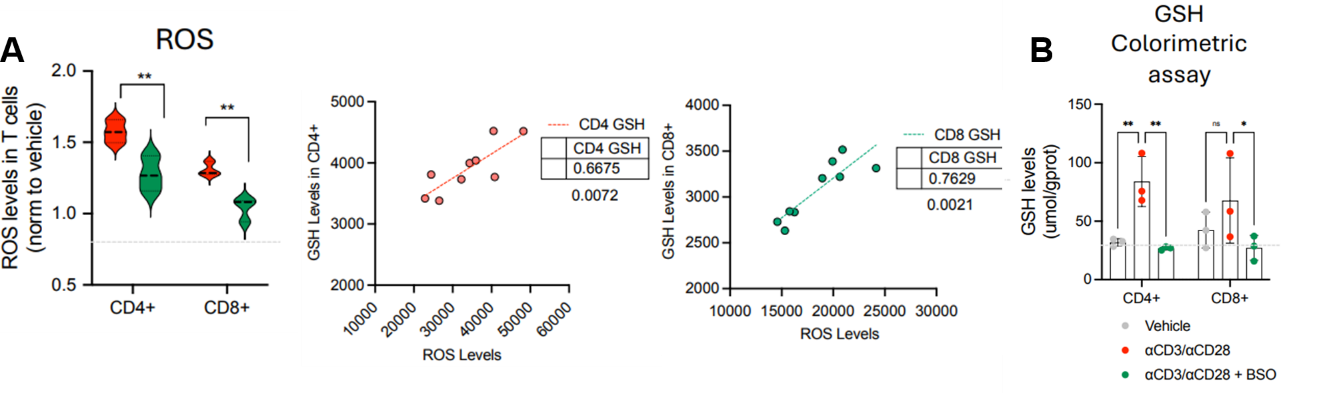


**Fig. S10.** **a (left)** Bar graph showing ROS levels in CD4⁺ and CD8⁺ T cells after 24 hours of stimulation with anti-CD3/CD28 alone (red) or in combination with BSO (2 mM, green) (mean ± S.E.M., n = 3 per group; two independent experiments; t-test, p < 0.01). **(Middle and right)** Correlation analysis between ROS and GSH levels measured in CD4⁺ (middle) and CD8⁺ (right) T cells. **b** Colorimetric assay of cytosolic GSH levels after 24 hours of treatment with anti-CD3/CD28 alone or in combination with BSO (2 mM) (mean ± S.E.M., n = 3 per group; three independent experiments) (mean ± S.E.M., n=3 per group, three independent experiments, ANOVA with post-hoc Tukey HSD test, * p<0.01).

**IX. GCL staining**

PBMCs were stimulated with TBHP (3.5 μM). PBMCs were stained with fluorescently conjugated antibodies (CD4 and CD8) and viability dye for 30 min. They were then fixed and permeabilized for additional 30 min, following which, cells were stained with fluorescent GCL antibody for 30 min. Cells were washed, resuspended in PBS and assessed by flow cytometry.

**X. Proliferation assay**

500,000 conventional PBMCs labeled with cell trace violet were treated with or without BSO (0.5 mM or 2 mM) and stimulated with αCD3/αCD28 for 3 days. They were then washed and stained with fluorescently conjugated antibodies (CD4 and CD8) and a viability dye for 30 min, and with or without **GLed** for 45 min. Cells were washed, resuspended in PBS and assessed by flow cytometry.


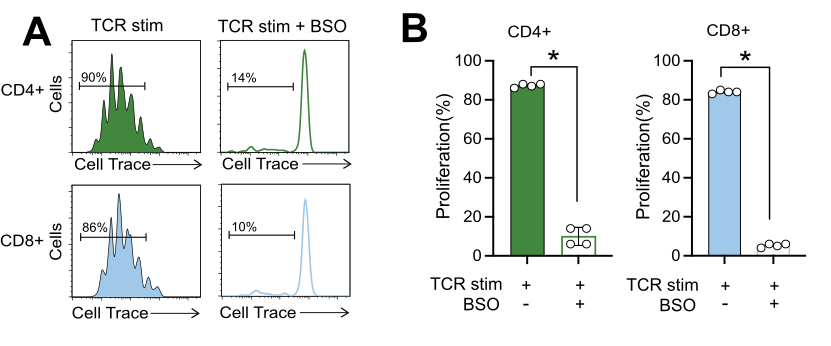


**Fig. S11. a** Representative MFIs from CD4^+^ and CD8^+^ T cell proliferation with and without BSO (2 mM) after TCR activation and the proliferation (%) **b** summarized in graph bars (mean ± S.E.M., n=4 per group, three independent experiments, t-test, *p<0.05).

**XI. Cytokine release**

For cytokine staining, 500,000 conventional PBMCs were stimulated with αCD3/αCD28 for 24h, adding a Golgi Plug (BD Biosciences) for the last 4 hours. Cells were then processed and stained with fluorescently conjugated antibodies (CD4 and CD8) and viability dye for 30 min, and with or without **GLed** for 45 min. They then were fixed and permeabilized for an additional 30 min, washed and stained with fluorescently IFNγ, GZMB, and IL-6 antibody for 30 min. Cells were washed, resuspended in PBS and assessed by flow cytometry.


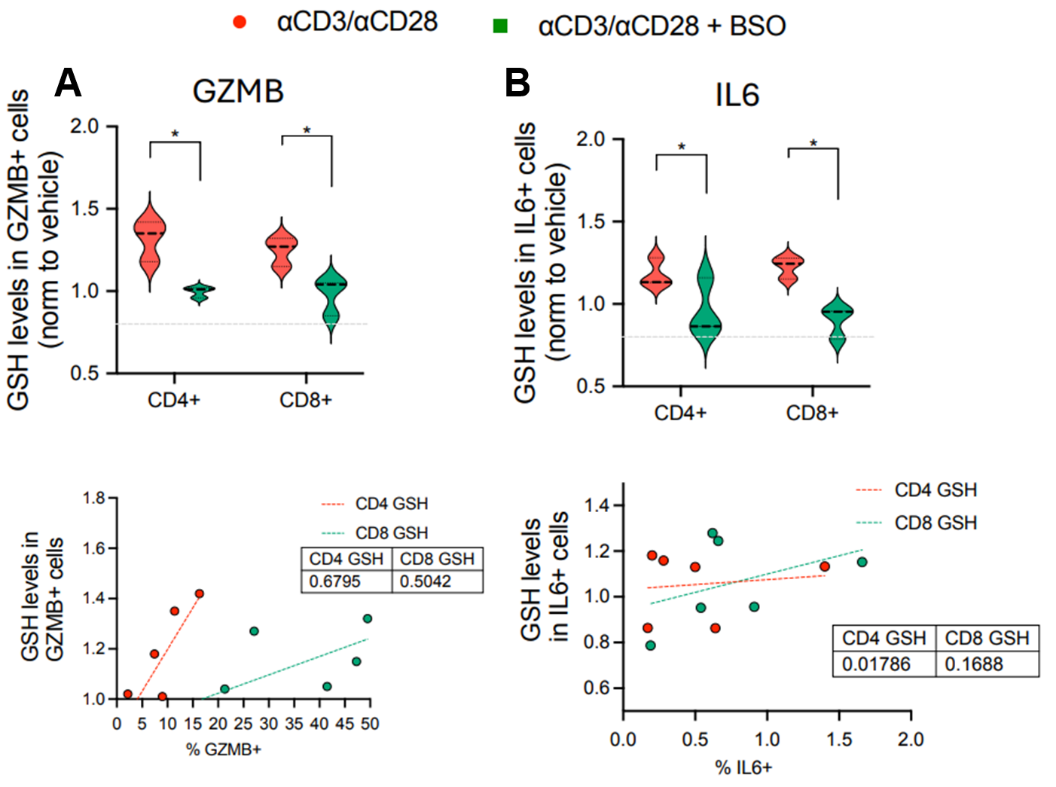


**Fig. S12. a** Bar graph showing GZMB expression levels in CD4⁺ and CD8⁺ T cells after 24 hours of stimulation with anti-CD3/CD28 antibodies ± BSO (2 mM). Below: correlation between GSH levels and GZMB expression in CD4⁺ and CD8⁺ T cells. between GSH levels and GZMB expression in CD4⁺ (middle) and CD8⁺ (right) T cells (mean ± S.E.M., n=3 per group, three independent experiments, t-test, *p<0.05). **b**, Same as in (a), but for IL-6 expression levels and their correlation with GSH in CD4⁺ and CD8⁺ T cells (mean ± S.E.M., n=3 per group, three independent experiments, t-test, *p<0.05).

**XII. Flow cytometry**

All samples were collected using **LSRFortessa X-20** or FACS Canto II flow cytometer (BD Biosciences) and analyzed using FCS Express 7 (De Novo Software), and Flowjo.

**XIII. Western Blot and Immunoprecipitation**

Pan T cells or previously sorted CD4⁺ or CD8⁺ T cells were harvested following treatment with various immunosuppressive drugs and TCR stimulation using αCD3/αCD28 for 48 hours. After centrifugation, the soluble fractions were collected, and protein concentration was determined using Pierce BCA protein assay (Thermo Scientific). Equal protein amounts were transferred to polyvinylidene difluoride membrane (Millipore) and probed with GCLc antibody. Immunocomplexes were detected using Immobilon Western Chemiluminescent horseradish peroxidase (HRP) Substrate (Millipore). Membranes were imaged using Li-Cor Odyssey Fc (LiCor Biosciences) and quantified using ImageJ.

## **XIV. GSH levels in the presence of immunosuppressive drugs**

**
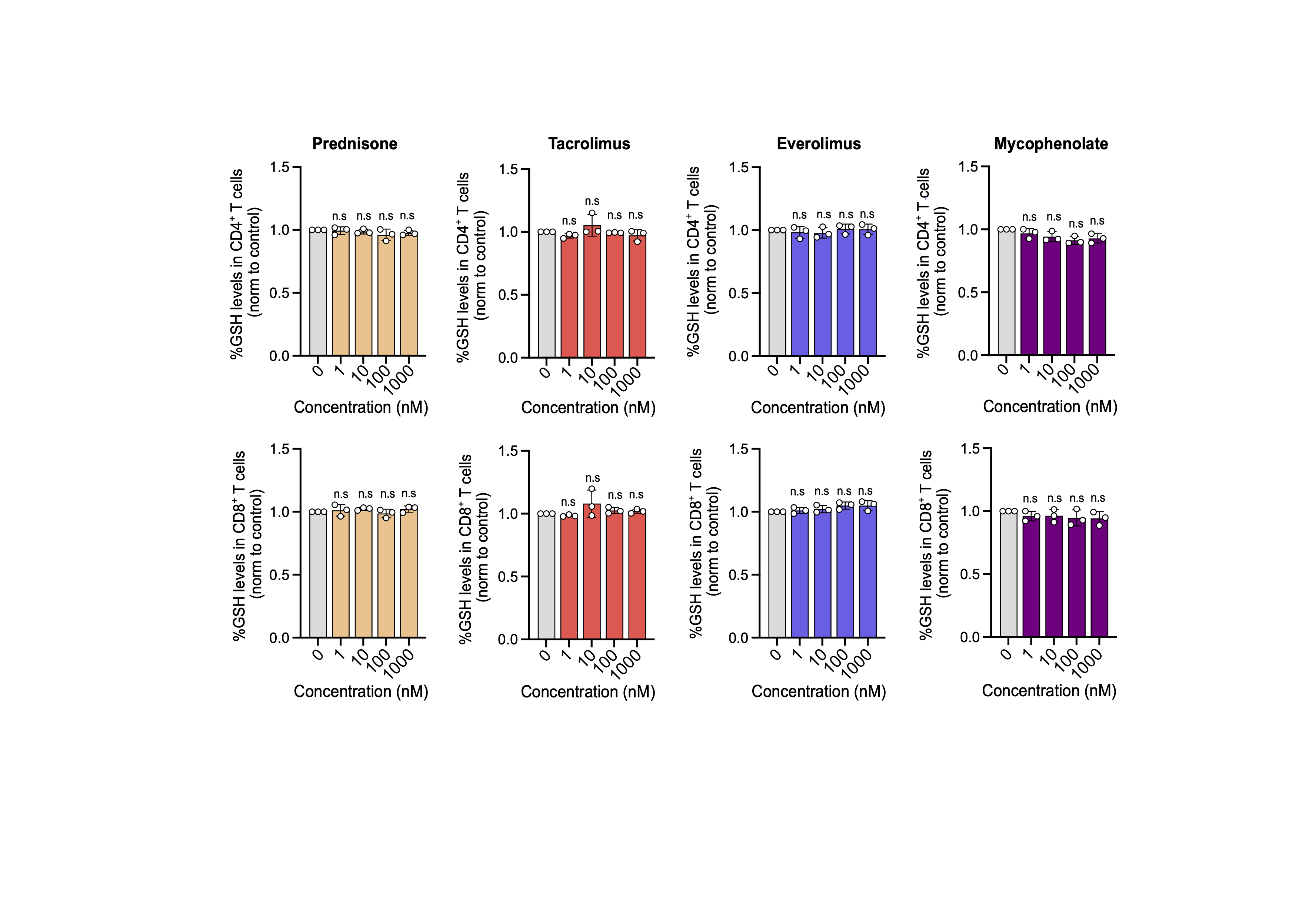
**

**Fig. S13.** Bar summaries of GSH levels in human CD4+ and CD8+ T cells after treatment with the indicated immunosuppressive drug for 24 hours without TCR activation normalized to control (vehicle) (mean ± S.E.M., n=4 per group, three independent experiments, ANOVA with Tukey HSD, n.s not significant).

**XV. NMR spectra (^1^H-NMR and ^13^C-NMR) of 3 and 4**

**
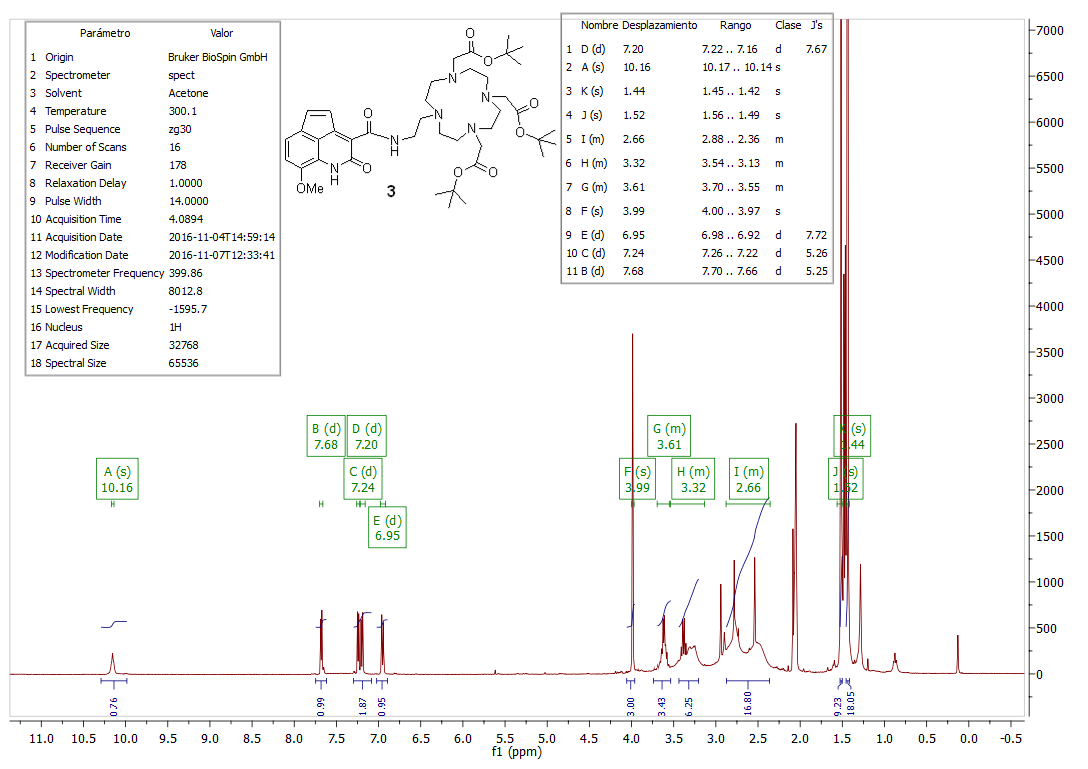
**
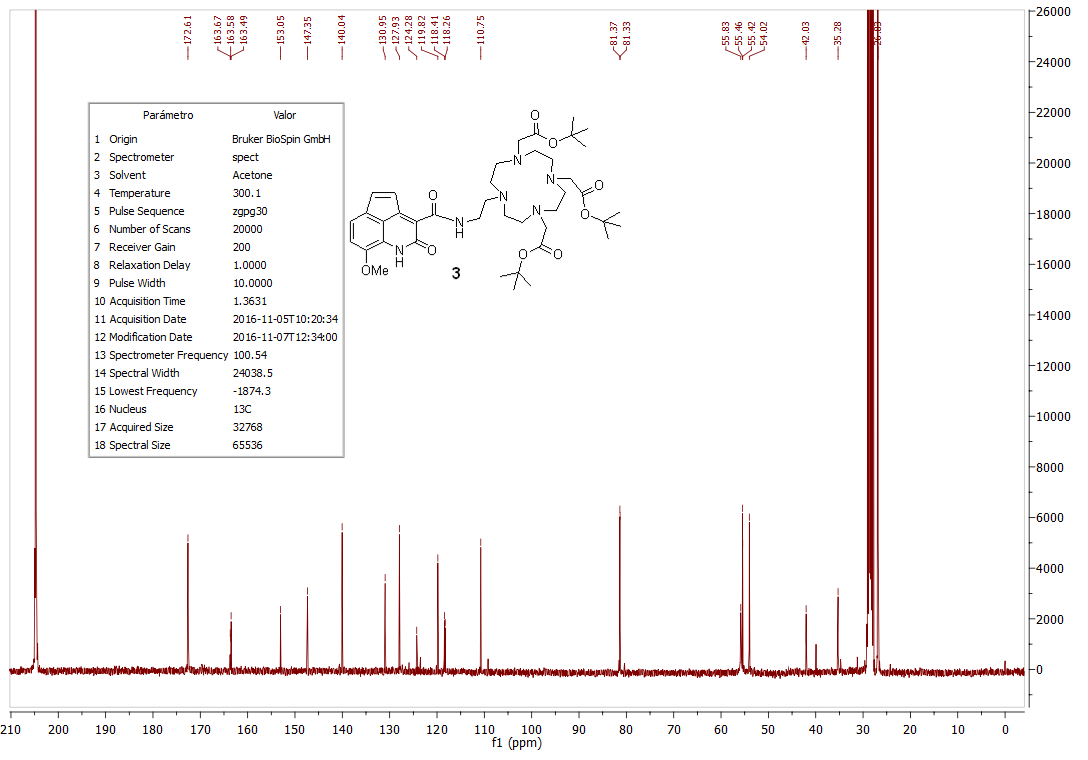


#### **Figure S7.** NMR spectra (^1^H, top; ^13^C, bottom) of **3**.


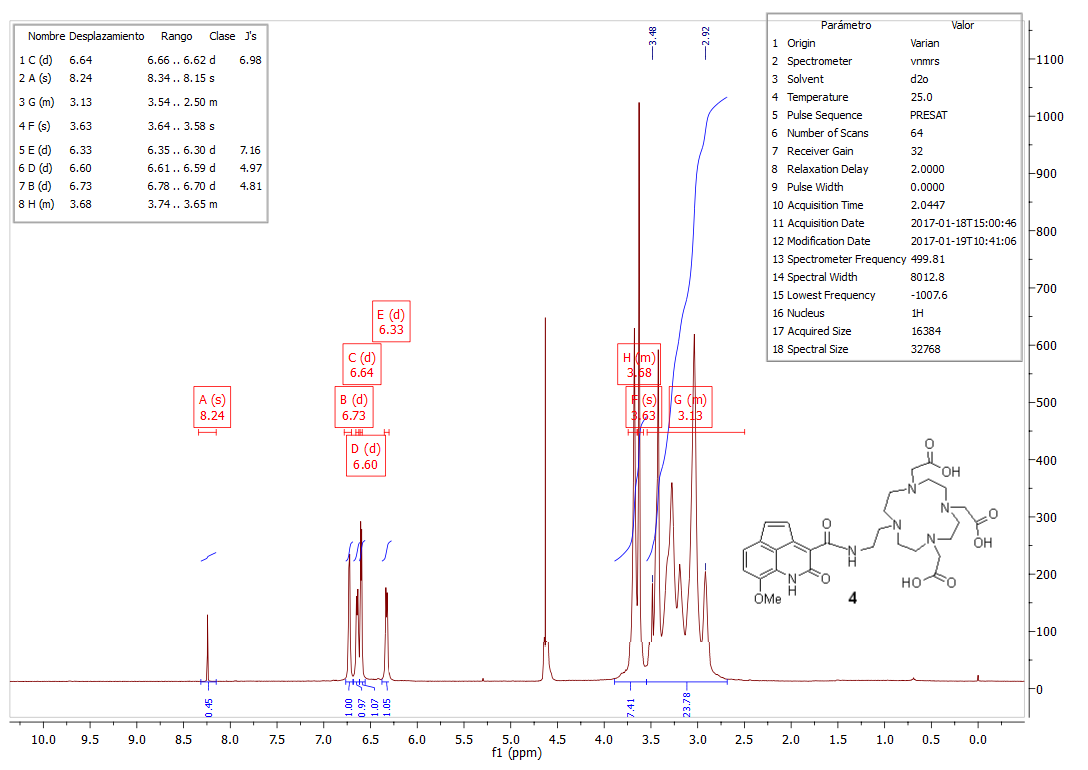


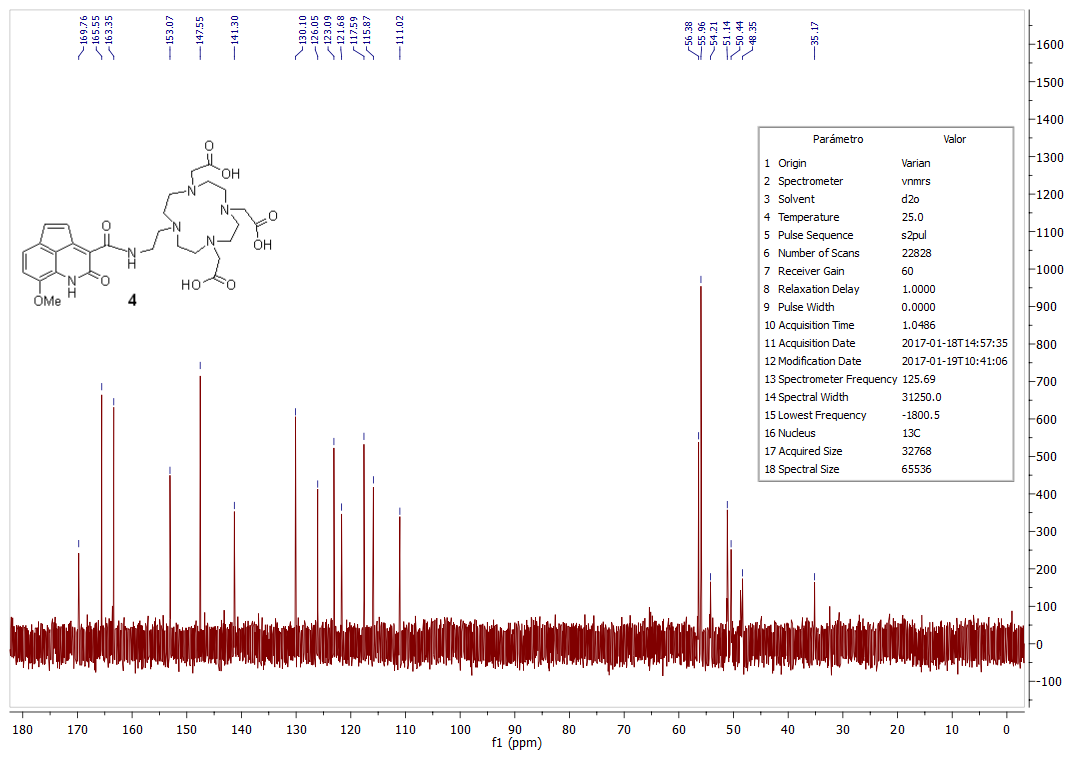


#### **Figure S8.** NMR spectra (^1^H, top; ^13^C, bottom) of **4**.

## **XVI. References**

1. Fueyo-González F*, et al.* Self-Assembled Lanthanide Antenna Glutathione Sensor for the Study of Immune Cells. *ACS Sensors* **7**, 322-330 (2022).

2. de la Reberdière A, Lachaud F, Chuburu F, Cadiou C, Lemercier G. Synthesis of a new family of protected 1,4,7,10-tetraazacyclododecane-1,4,7-triacetic acid derivatives with thioctic acid pending arms. *Tetrahedron Letters* **53**, 6115-6118 (2012).

3. Buschmann V, Orthaus S, Devaux A, Erdmann R. Phosphorescence Lifetime Imaging Microscopy (PLIM) Measurements: Practical Aspects. *Technical Note, Picoquant GmbH*, (2012).

4. Schindelin J*, et al.* Fiji: an open-source platform for biological-image analysis. *Nat Meth* **9**, 676-682 (2012).

5. van Meerloo J, Kaspers GJ, Cloos J. Cell sensitivity assays: the MTT assay. *Methods Mol Biol* **731**, 237-245 (2011).
